# Research advances in the structures and biological activities of secondary metabolites from *Talaromyces*

**DOI:** 10.3389/fmicb.2022.984801

**Published:** 2022-08-19

**Authors:** Li-Rong Lei, Lei-Qiang Gong, Meng-Ying Jin, Rui Wang, Ran Liu, Jing Gao, Meng-Dan Liu, Li Huang, Guang-Zhi Wang, Dong Wang, Yun Deng

**Affiliations:** State Key Laboratory of Characteristic Chinese Medicine Resource of Southwest China, School of Pharmacy, Chengdu University of Traditional Chinese Medicine, Chengdu, China

**Keywords:** *Talaromyces*, secondary metabolite, biological activity, polyketides, terpenoids, nitrogen compounds

## Abstract

The genus *Talaromyces* belongs to the phylum Ascomycota of the kingdom Fungi. Studies have shown that *Talaromyces* species yield many kinds of secondary metabolites, including esters, terpenes, steroids, alkaloids, polyketides, and anthraquinones, some of which have biological activities such as anti-inflammatory, bacteriostatic, and antitumor activities. The chemical constituents of fungi belonging to the genus *Talaromyces* that have been studied by researchers over the past several years, as well as their biological activities, are reviewed here to provide a reference for the development of high-value natural products and innovative uses of these resources.

## Introduction

As new diseases have emerged in recent years in response to environmental changes, the search for new sources to develop effective and safe drugs cannot be delayed. Natural resources offer the potential to find new structural classes with unique bioactivities for disease treatment. Endophytic fungi represent a rich source of bioactive metabolites (Uzma et al., 2018). The genus *Talaromyces* is widely distributed in soil, plants, sponges, and foods. Recent findings have demonstrated that *Talaromyces* are very abundant in marine environments (Nicoletti and Vinale, 2018). This may be due to the fact that the ocean itself is rich in species resources. Moreover, the extreme living conditions of the oceans have led marine microorganisms to develop more specific metabolic patterns and *Talaromyces* can produce a number of structurally diverse active substances. Their metabolites have a wide range of biological activities, such as anti-inflammatory meroterpenoids, thioester-containing benzoate derivatives that exhibit significant α-glucosidase inhibitory activity and oxaphenalenone dimers with broad antibacterial activity. In this paper, we will summarize and describe the research on the secondary metabolites of *Talaromyces* species and their biological activities over the past several years, to provide a reference for subsequent research on *Talaromyces*, and to provide an outlook on the problems in the isolation and analysis of fungal secondary metabolites and the prospect of *Talaromyces* species. The current problems in the isolation and analysis of fungal secondary metabolites are summarized and the prospects of their utilization are provided.

## Research status of *Talaromyces* species

**Figure 1:**
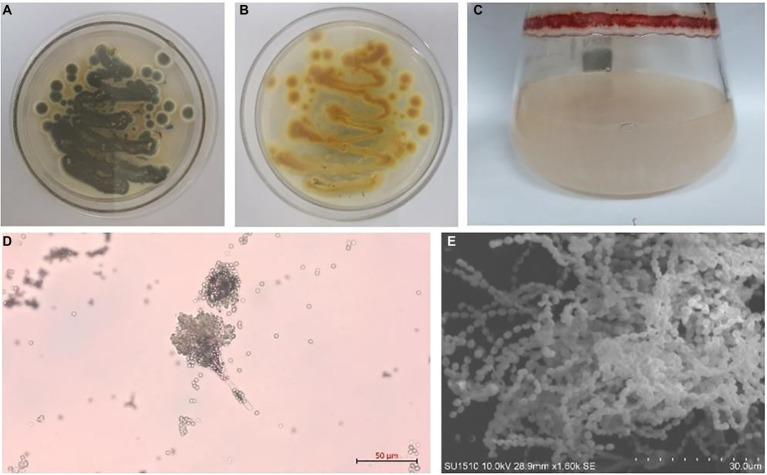
*Talaromyces amestolkiae* (CBS 365.48) *in vitro*. **(A,B)** Growth of *T. amestolkiae* on M1 semisolid medium at 30°C after 7 d and **(C)** in liquid M1 medium at 30°C after 7 d; **(D)** conidia, scale bar = 10 μm; **(E)** *T. amestolkiae*, SEM.

**Figure 2:**
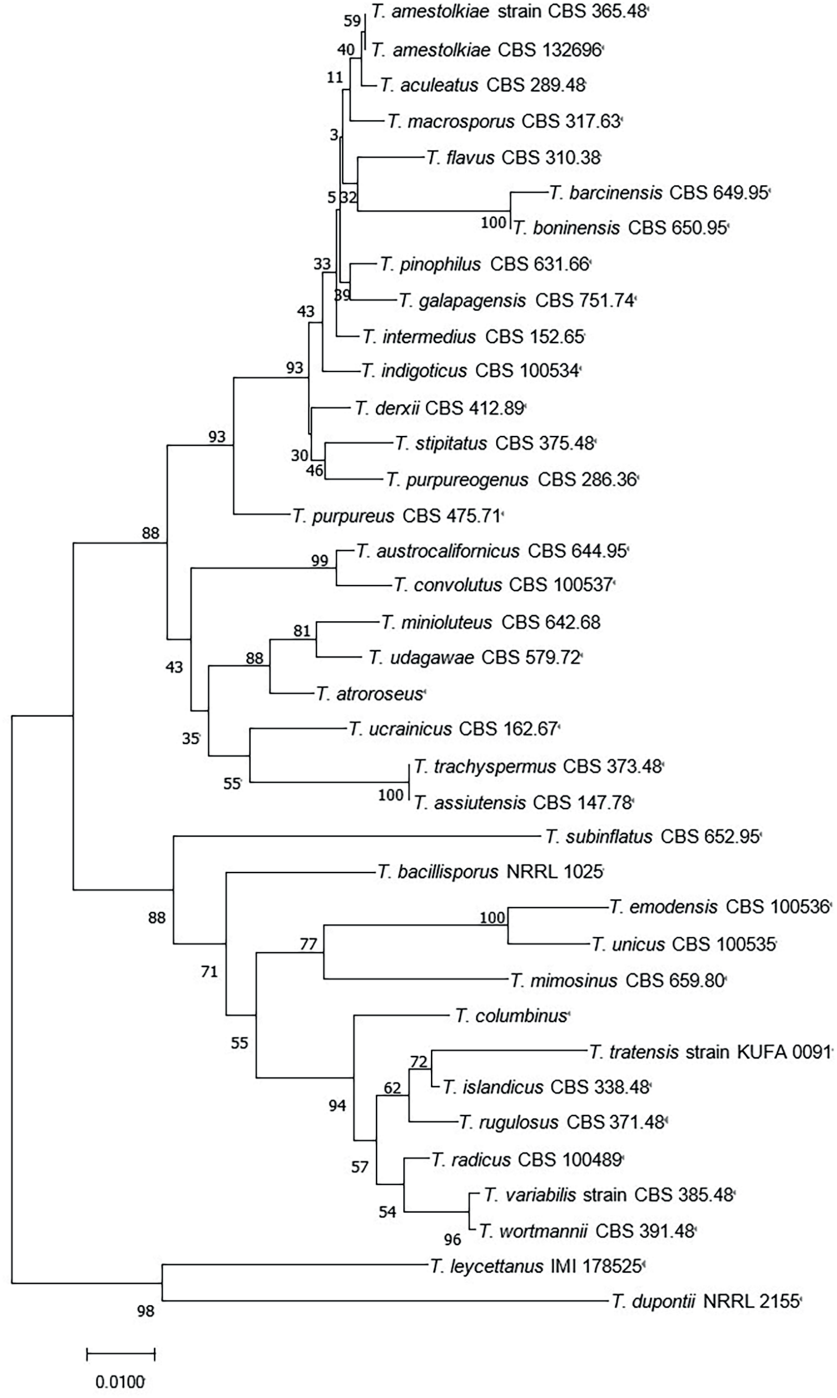
Neighbor-joining tree of *T. amestolkiae* (CBS 365.48) based on ITS rDNA.

*Talaromyces* belongs to the fungal phylum, ascomycete subphylum, ascomycetes, sporangia, and fungal family, which are widely distributed in sponges, plants, and soil. The colonies started out yellow and slowly turned gray-green over the course of a week. The middle of the back is yellow, and the edges are white (Figure 1). *Talaromyces* has various species (Figure 2). *T. marneffei*, *T. funiculosum*, and *T. purpureogenus* are the most studied strains at present. In addition, new strains, such as *T. rubrifaciens*, *T. australis*, *T. kendrickii*, *T. veerkampii*, *T. fuscoviridis*, and *T. stellenboschiensi* were isolated and purified (Visagie et al., 2015; Luo et al., 2016), and the corresponding chemical constituents were studied, which greatly enriched the species of chemical constituents of the fungi. The secondary metabolites of *Talaromyces* are rich in species, have novel structures and have good biological activity, which provides a basis for the development and application of endophytes. At present, the compounds isolated from the secondary metabolites of *Talaromyces* include esters, terpenoids and steroids, alkaloids, polyketones, anthraquinones and others, and most of them have good biological activities such as anti-inflammatory, antibacterial and antitumor activities.

Related studies have shown that *Talaromyces* species have great potential in agriculture, food, cosmetics, medicine, and environmental protection. In the field of agriculture, *Talaromyces* species can inhibit pathological changes in crops and promote crop growth. *T. tratensis* can be used as a biological control agent to control brown spot and dirty panicle diseases in rice (Dethoup et al., 2018). The secondary metabolites in *T. tratensis*, such as glucanase, can effectively treat rot disease that affects the yield of cucumbers and tomatoes (Halo et al., 2019). *T. flavus* not only promotes the growth of cotton and potatoes (Naraghi et al., 2012) but also produces an enzyme that plays an important role in resisting plant diseases for their strong capacity of degrading chitin (Xian et al., 2011). Most *Talaromyces* species can produce a red pigment (Frisvad et al., 2013; Venkatachalam et al., 2018), which can be used as a natural colorant in cosmetics and foods. The thermostable enzyme produced in *T. emersonii* can effectively improve bread quality with respect to hardness, staling, and loaf volume (Waters et al., 2010). An aspartic protease from *T. leycettanus* has strong proteolytic activity and improves the clarity of fruit juice (Guo et al., 2019). *Talaromyces* species can produce many other bioactive secondary metabolites, and these compounds have been found to have antibacterial, anti-inflammatory, antitumor, antioxidant, nematocidal, and other effects in medical research. Secondary metabolites from an Australian Marine Tunicate-Associated Fungus *Talaromyces* sp. (CMB-TU011) exhibit certain antibacterial activities (Dewapriya et al., 2018). GH3 β-glucosidases from *T. amestolkiae* expressed in *Pichia pastoris* can transglycosylate phenolic molecules, and the resulting transglycosylation products can improve the biological activity of the original aglycones against breast cancer cells (Méndez-Líter et al., 2019). Talaraculones from a strain of *T. aculeatus* can inhibit the activity of α-glucosidase and can be used to prevent the progression of type II diabetes, as well as for the early treatment of type II diabetes (Ren et al., 2017). In the field of environmental protection, biosorption by microorganisms has been proven to be an effective technique for removing heavy metals from wastewater. A biological adsorbent formed by combining *T. amestolkiae* with a specific chitosan sponge can effectively remove trace heavy metals or high concentrations of lead from industrial wastewater (Wang et al., 2019). *Talaromyces* sp. KM-31 can remove arsenic from heavily polluted wastewater and can thus be employed in bioremediation strategies (Nam et al., 2019).

According to the classification of the chemical components, this paper will summarize and explain research carried out on secondary metabolites from *Talaromyces* species and their biological activities over the past 10 years with the aim of providing references for follow-up studies of *Talaromyces*, at the same time, the problems existing in the separation and analysis of fungal secondary metabolites and the prospect of *Talaromyces* species, as well as summarizing existing problems in the separation and analysis of fungal secondary metabolites and prospects for the use of secondary metabolites from *Talaromyces* species.

## Studies on the chemical constituents and activity of *Talaromyces*

### Ester-based compounds

#### Esters

**Figure 3:**
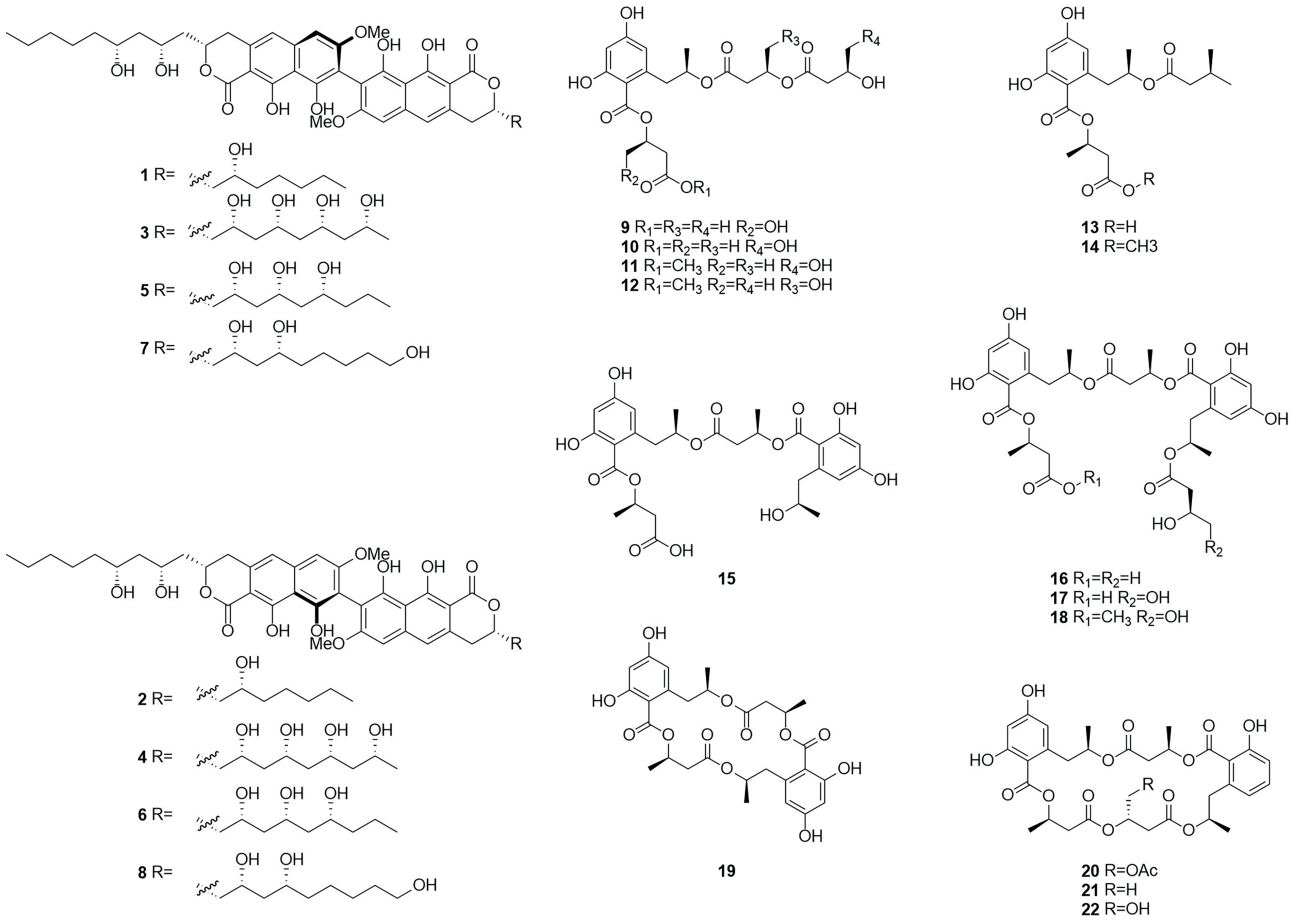
Chemical structures of compounds **1–22**.

Esters are chemical compounds derived by reacting an oxoacid with a hydroxyl compound such as an alcohol or phenol (Sparkman et al., 2011). Dinapinones AB1 and AB2 (**1** and **2**), dinapinones AC1 and AC2 (**3** and **4**), dinapinones AD1 and AD2 (**5** and **6**), and dinapinones AE1 and AE2 (**7** and **8**; Figure 3), which were isolated from the fermentation broth of *T. pinophilus* FKI-3864 in 2013 (Kawaguchi et al., 2013), were identified and characterized as ester derivatives. These dinaphthoquinones have the same backbone of aryl dihydronaphthoquinone and consist of one monapinone A and one different monapinone in a heterodimer. Compound **2** had a strong inhibitory effect on triacylglycerol synthesis in intact mammalian cells, with an IC_50_ value of 1.17 μM.

**Figure 4:**
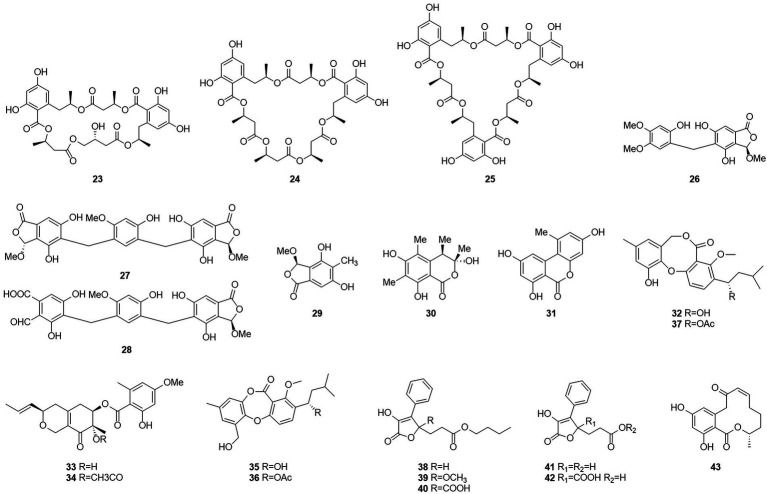
Chemical structures of compounds **23–43**.

Seventeen new polyesters were isolated from the fermentation products of the wetland soil-derived fungus *T. flavus*, namely, talapolyesters A–F (**9**–**12**, **22** and **24**), 15G256ν (**13**), 15G256ν-me (**14**), 15G256π (**15**), 15G256β-2 (**16**), 15G256α-2 (**17**), 15G256α-2-me (**18**), 15G256ι (**19**), 15G256β (**20**), 15G256α (**21**), 15G256α-1 (**23**) (Figure 4), and 15G256ω (**25**) (He et al., 2014b). All macrocyclic polyesters (**19**–**25**) were cytotoxic to HL-60, SMMC-7721, A-549, MCF-7, and SW480 tumor cells, while linear polyesters (**9**–**18**) were inactive with IC_50_ > 40 mM compared to cisplatin. This suggests that a macrocyclic structure is required for cytotoxicity. Among them, **20** and **25** showed significant cytotoxic activity against MCF-7 cell lines with IC_50_ of 3.27 and 4.32 μM, respectively. The cytotoxic activity of 15G256 polyester was systematically investigated for the first time and a tight conformational relationship is presented.

Talaromycolides A–C (**26**–**28**), rubralide C (**29**), sclerotinin A (**30**), alternariol (**31**), and penicillide (**32**) were obtained from the epiphytic fungal strain *T. pinophilus* AF-02, which was isolated from green Chinese onion, in 2015 (Zhai et al., 2015). Compound **26** [minimum inhibitory concentration (MIC) = 12.5 μg/ml] showed stronger inhibitory activity against *Clostridium perfringens* than erythromycin, streptomycin, acheomycin, and ampicillin. Compound **26** (MIC = 6.25 μg/ml) showed similar inhibitory activity to acheomycin and was superior to levofloxacin, ampicillin, and streptomycin against *Bacillus subtilis*. Compound **27** (MIC = 12.5 μg/ml) showed higher inhibitory activity than erythromycin and ampicillin against *Bacillus megaterium* and higher inhibitory activity than erythromycin, ampicillin, and streptomycin against *Escherichia coli* (MIC = 25 μg/ml). Compound **28** (MIC = 25 μg/ml) was more active against *C. perfringens* than erythromycin, streptomycin, acheomycin and ampicillin.

In 2015, the structures of compounds **33** and **34** were characterized as deacetylisowortmins A and B, which were isolated from *T. wortmannii* LGT-4 derived from the leaves of a mangrove plant *Acanthus ilicifolius* (Fu et al., 2016). Four esters, talaromyones A and B (**35** and **36**), penicillide (**32**), and purpactin A (**37**), were obtained from a fermentation product of the mangrove endophytic fungus *T. stipitatus* SK-4 in 2016 (Cai et al., 2017). Compound **36** exhibited antibacterial activity against *B. subtilis* with an MIC value of 12.5 μg/ml. In the α-glucosidase inhibition assay, compounds **36** and **37** showed some inhibitory activity with an IC_50_ values of 48.4–99.8 μM.

**Figure 5:**
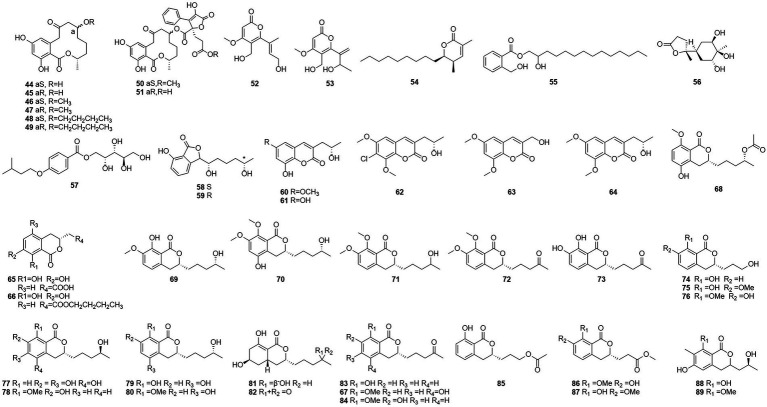
Chemical structures of compounds **44–89**.

Five butenolides (**38**–**42**), seven (3S)-resorcylide derivatives (**43**–**49**) (Figure 5), two butenolide-resorcylide dimers (**50** and **51**) were yielded by culture on a solid rice medium of *T. rugulosus* isolated from the Mediterranean sponge *Axinella cannabina* (Küppers et al., 2017). The butenolide–resorcylide dimers talarodilactones A and B (**50** and **51**) was highly cytotoxic to the L5178Y mouse lymphoma cell line with IC_50_ of 3.9 μM and 1.3 μM, respectively.

Talaromycin A (**52**) and clearanol A (**53**) were isolated from the endophytic fungus *Talaromyces* sp. MH551540 associated with *Xanthoparmelia angustiphylla* in 2018 (Yuan et al., 2018). Compound **52** and **53** had selective cytotoxicity against MDA-MB-231 cells. Compound **54**, which was identified as wortmannine F, was obtained from cultures of the endophytic fungus *T. wortmannii* LGT-4 isolated from *Tripterygium wilfordii* and has a strong phosphoinositide-3-kinase-α (PI3K-α) inhibitory activity with an IC_50_ value of 25 μM (Zhao et al., 2019b). Pentalsamonin (**55**) was isolated from submerged fermentation on Bengal gram husk (BegH) of *T. purpureogenus* CFRM-02 (Pandit et al., 2018). The MIC and MBC of pentalsamonin (**55**) against *B. subtilis*, *Staphylococcus aureus*, *E. coli*, and *Klebsiella pneumoniae* were 62.5–125 and 125–250 μg/ml, respectively.

Talaromarnine A (**56**) and talaromarnine B (**57**) were obtained from cultures of *T. marneffei*, an endophytic fungus of *Epilobium angustifolium* (Yang et al., 2021). Two previously undescribed phthalides, amestolkins A (**58**) and B (**59**) were isolated from *T. amestolkiae* derived from *Syngnathus acus* Linnaeus in Lingshui Li Autonomous County, Hainan Province, China, which has the same planar structure of (1,5-dihydroxyhexyl)-7-hydroxyisobenzofuran-1(3H)-one. They were shown to inhibit gene expressions of proinflammatory factors including C-C motif chemokine ligand 2 (CCL-2), tumor necrosis factor-α (TNF-α), and interleukin-6 (IL-6) as well as reducing the secretion of inducible nitric oxide synthase (iNOS) in BV2 microglia at the concentration of 30 μM (Huang et al., 2022).

#### Coumarins

Coumarinic compounds are lactones resulting from the fusion of a benzene ring and a α-pyrone ring (Batista et al., 2021). Talacoumarins A and B (**60** and **61**), which were characterized as coumarins, were isolated from the fermentation broth of the wetland soil fungus *T. flavus* (He et al., 2014c). Activity tests showed that compounds **60** and **61** exhibited moderate activity against the aggregation of Aβ42. This was the first report to state that a coumarin can inhibit Aβ42 aggregation. A new compound **62**, chloropestalasin A was isolated from *T. amestolkiae* derived from submerged wood collected from fresh water, along with 3-hydroxymethyl-6,8-dimethoxycoumarin (**63**) and pestalasin A (**64**) (El-Elimat et al., 2021).

#### Isocoumarin

Isocoumarin is the common name for 1H-2-benzopyran-1-one skeleton (Braca et al., 2012). Three dihydroisocoumarins (**65**–**67**) were yielded by culture on a solid rice medium of *T. rugulosus* isolated from the Mediterranean sponge *Axinella cannabina* (Küppers et al., 2017). Six new isocoumarin derivatives, talaromarins A-F (**68**–**73**), and 17 known analogues (**67**, **74**–**89**), were isolated from the mangrove-derived fungus *T. flavus* (Eurotiales: Trichocomaceae) TGGP35 (Cai et al., 2022). Compounds **67**, **73**–**78**, **84**–**85** and **87**–**89** showed similar or better IC_50_ values for antioxidant activity ranged from 0.009 mM to 0.27 mM, compared to the positive control trolox (IC_50_ = 0.29 mM). Compounds **77**, **84**, **87** and **89** showed strong inhibitory activity. IC_50_ values of 0.10 ~ 0.62 mM against α-glucosidase and 0.5 mM for the positive control acarbose activity at 50 μg/ml and 1 mg/ml concentrations. These results suggest that isocoumarins have important applications in the development of antioxidants and in the control of diabetes mellitus. Talaroisocoumarin A (**73**) was obtained from marine-derived *Talaromyces* sp. ZZ1616 in potato dextrose broth medium. The MIC values of talaroisocoumarin A against methicillin-resistant *S. aureus*, *E. coli* and *Candida albicans* were 36.0 μg/ml, 32.0 μg/ml and 26.0 μg/ml, respectively (Ma et al., 2022).

### Polyketones

**Figure 6:**
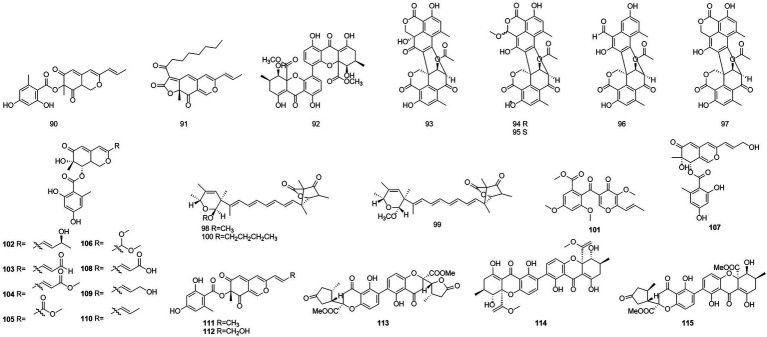
Chemical structures of compounds **90–115**.

Polyketides were named in the 1890s to refer to a structurally diverse group of natural products that contained many carbonyls and alcohols, generally separated by methylene carbons. They are synthesized by a series of decarboxylative condensation reactions between small carboxylic acids and malonate using polyketide synthases (PKSs; Richardson and Khosla, 1999). Two polyketones, mitorubrin (**90**) and monascorubrin (**91**) (Figure 6), were isolated from *T. atroroseus* (Frisvad et al., 2013). Because no citrinin was found in any *Talaromyces* species, it may be a good alternative for red pigment production. Compound **92**, which was characterized as a polyketone and named talaroxanthone, was obtained from the fermentation products of an endophytic strain of a *Talaromyces* sp. isolated from the Amazonian rainforest plant *Duguetia stelechantha* root (Koolen et al., 2013). Five compounds, 9a-epi-bacillisporin E (**93**), 1-epi-bacillisporin *F* (**94**), and bacillisporins F-H (**95**–**97**) were isolated from the fermentation products of the soil fungus *T. stipitatus* (Zang et al., 2016). Compound **97** exhibited some antibacterial activity and some cytotoxicity against HeLa cells. Compounds **98**–**100**, wortmannilactones I1-I3, which were identified and characterized as three new polyketides, were purified from *T. wortmannii* using the one strain–many compounds strategy. These compounds showed selective inhibitory activity against NADH fumarate reductase (Liu et al., 2016).

**Figure 7:**
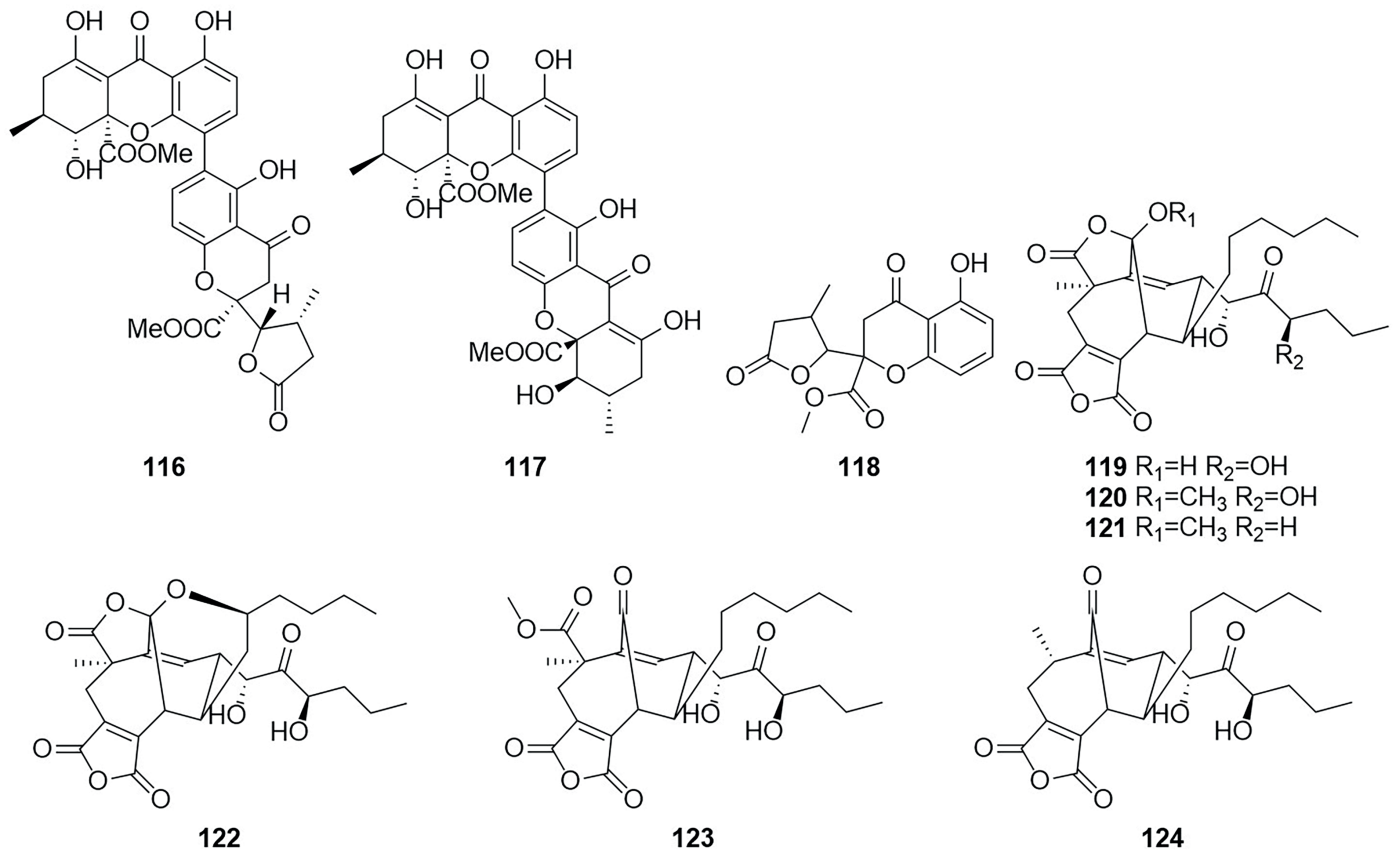
Chemical structures of compounds **116–124**.

The polyketone 3-O-methylfunicone (**101**) was isolated from the culture filtrate of an endophytic strain of *T. pinophilus* obtained from the strawberry tree (*Arbutus unedo*) in 2017 (Vinale et al., 2017). On water agar at a concentration of 0.1 mg/ml, it completely inhibited the growth of phytopathogenic fungi such as *Rhizoctonia solani* (De Stefano et al., 1999). Eleven polyketones, talaraculones A–F (**102**–**107**), pinazaphilone B (**108**), pinophilin B (**109**), Sch 725680 (**110**), (−)-mitorubrin (**111**), and (−)-mitorubrinol (**112**), were obtained from the fungus *T. aculeatus*, which was isolated from saline-alkali soil (Ren et al., 2017). The results of the activity tests showed that compounds **102** and **103** exhibited very high levels of inhibitory activity against α-glucosidase than the positive control acarbose (IC_50_ = 101.5 μM), with IC_50_ values of 78.6 and 22.9 μM, respectively. Compounds that were defined and characterized as six polyketones, paecillin D (**113**), secalonic acid A (**114**), blennolide G (**115**), versixanthone A (**116**) (Figure 7), penicillixanthone A (**117**), and paecillin B (**118**), were isolated from the fermentation products of three Amazonian plants endophytic strains of *T. stipitatus* in 2018 (da Silva et al., 2017). Activity tests showed that compounds **113** and **116** were active against yeasts (MICs of 15.6 μg/ml and 31.3 μg/ml, respectively).

Six new nonadride derivatives, named talarodrides A–F (**119–124**), were isolated from the antarctic sponge-derived fungus *Talaromyces* sp. HDN1820200. Talarodride A (**119**) and talarodride B (**120**) showed selective inhibitory effects against *Proteus mirabilis* and *Vibrio parahemolyticus* with MICs of 3.13–12.5 μM (Zhao et al., 2021b).

### Anthraquinone

**Figure 8:**
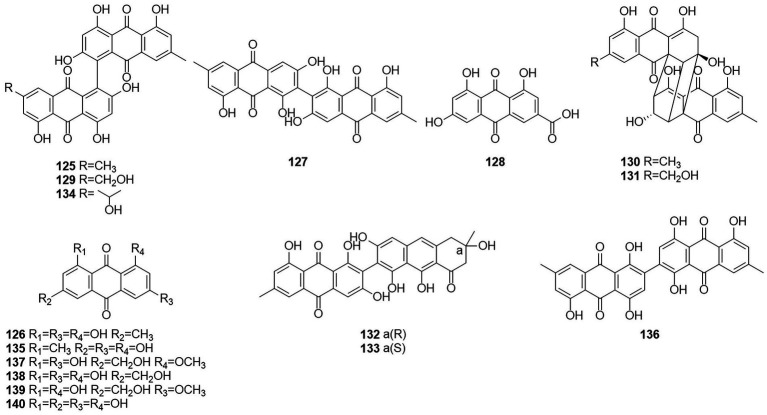
Chemical structures of compounds **125–140**.

Anthraquinones (AQs) are derived from anthracenes and have two keto groups, mostly in positions 9 and 10. The basal compound, anthraquinone (9,10-dioxoanthracene), can be substituted in various ways, resulting in a great diversity of structures (Vasil et al., 1984). Two anthraquinone compounds skyrin (**125**) and emodin (**126**) (Figure 8) were obtained from an extract of the mangrove endophytic fungus *Talaromyces* sp. ZH-154, which was isolated from the stem bark of *Kandelia candel* (Liu et al., 2010). Both compounds exhibited moderate cytotoxic activity against KB and KBv200 cells. The anthraquinone monomer (**126**) showed higher bioactivity than the dimer dianthraquinone (**125**). A new anthraquinones biemodin (**127**) and five known anthraquinones emodic acid (**128**), skyrin (**125**), oxyskyrin (**129**), and rugulosins A and B (**130** and **131**) were isolated from cultures of the endophytic fungus *T. wortmannii* obtained from healthy inner tissues of *Aloe vera* (Bara et al., 2013a). In the same year, two anthraquinone compounds, talaromannins A and B (**132** and **133**), were obtained from *T. wortmannii* in *A. vera* (Bara et al., 2013b). Both compounds displayed moderate MICs in a comparable concentration range for *S. aureus* and **132** represented the most active congeners.

Five anthraquinones were isolated from the solid fermentation products of the endophytic fungus *Talaromyces* sp. YE3016 (Xie et al., 2016). These compounds were 3-demethyl-3-(2-hydroxypropyl)-skyrin (**134**), skyrin (**125**), oxyskyrin (**129**), emodin (**126**), and 1,3,6-trihydroxy-8-methylanthraquinone (**135**). Activity tests showed that compounds **134**, **125**, and **129** displayed moderate cytotoxic activity against the MCF-7 cell line. Six anthraquinone compounds, 2,2′-bis-(7-methyl-1,4,5-trihydroxy-anthracene-9,10-dione) (**136**), emodin (**126**), questinol (**137**), citreorosein (**138**), fallacinol (**139**), and rheoemodin (**140**), were obtained from an ethyl acetate extract of a culture of the fungus *T. stipitatus* KUFA 0207, which is derived with a marine sponge (Noinart et al., 2017). Emodin (**126**), questinol (**137**), citreorosein (**138**), fallacinol (**139**), and rheoemodin (**140**) were tested for their anti-obesity activity using the zebrafish Nile red assay. The results showed that only the anthraquinones questinol (**137**) and citreorosein (**138**) had significant anti-obesity activity. Questinol (**137**) and citreorosein (**138**) reduced >60% and > 90% of the stained lipids with the IC_50_ values of 0.95 and 0.17 μM, respectively. The positive control resveratrol (REV) had an IC_50_ value of 0.6 μM. Emodin (**140**) caused toxicity (death) for all exposed zebrafish larvae after 24 h, while fallacinol (**139**) and rheoemodin (**140**) did not have any significant effects. It is interesting to observe that questinol (**137**), citreorosein (**138**) and fallacinol (**139**) are structurally similar, all having a hydroxymethyl group on C-6 and a hydroxyl group on C-8. Replacing the hydroxyl group on C-1 by a methoxyl group, as in questinol (**137**), diminishes the activity whereas replacing the hydroxyl group on C-3 with a methoxyl group, as in fallacinol (**139**), completely removes the anti-obesity activity. Therefore, it seems that the hydroxymethyl group on C-6 and the hydroxyl groups on C-3 and C-8 are necessary for the anti-obesity activity of the polyhydroxy anthraquinones.

### Terpenoids

**Figure 9:**
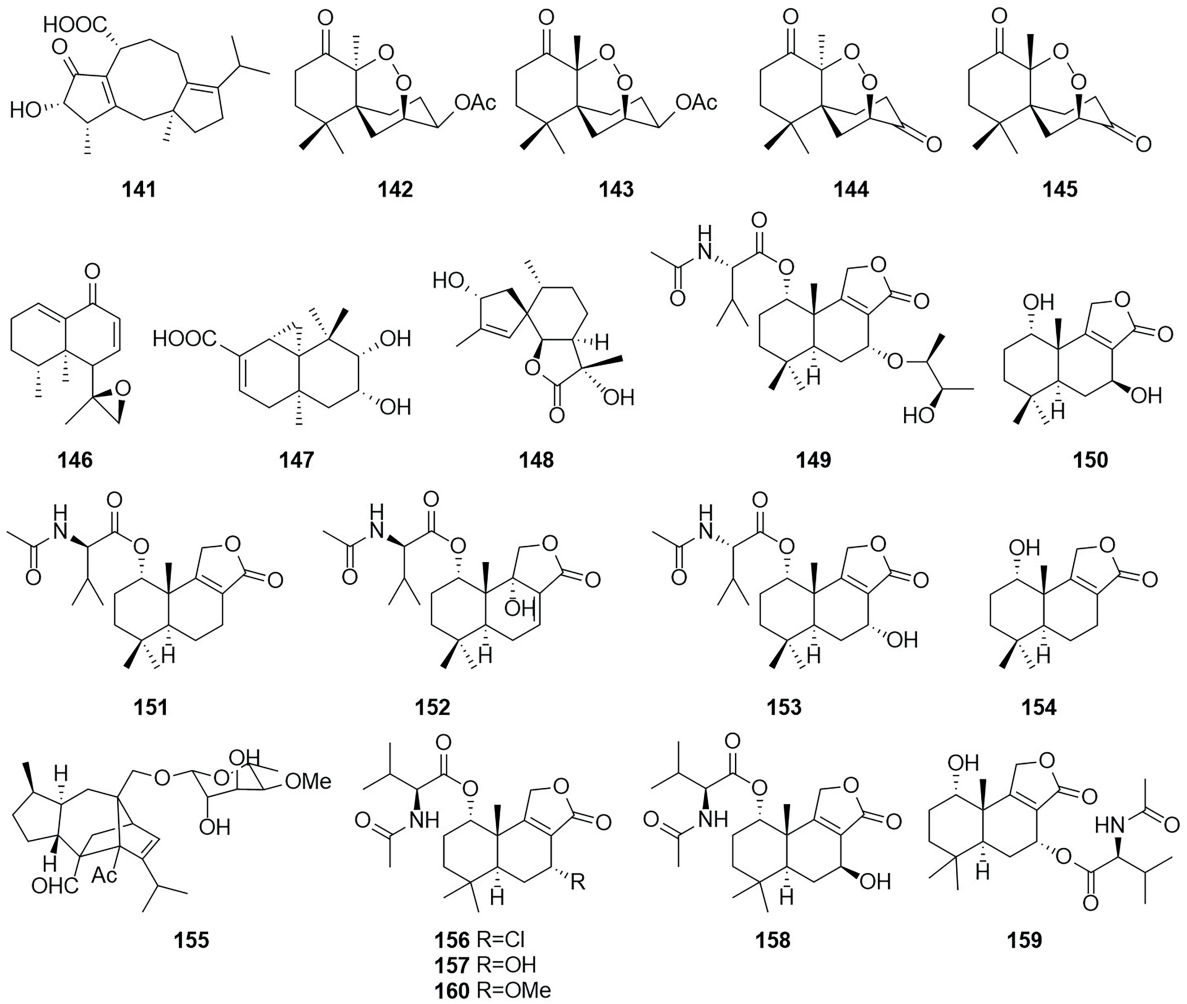
Chemical structures of compounds **141–160**.

Terpenoids otherwise known as isoprenoids are a large and diverse class of naturally occurring compounds derived from five carbon isoprene units (Reyes et al., 2018). Terpenoids are classified as hemiterpenes (C5), monoterpenes (C10), sesquiterpenes (C15), diterpenes (C20), sesterterpenes (C25), triterpenes (C30), and tetraterpenes/carotenoids (C40) (Adefegha et al., 2022). Compound **141** (Figure 9), which was characterized as a new fusicoccane diterpene and named pinophicin A, was obtained from the endophytic fungus *T. pinophilus* collected from the aerial parts of *Salvia miltiorrhiza* in 2019 (Zhao et al., 2021a). Four new sesquiterpene peroxides, talaperoxides A–D (**142**–**145**), were isolated from the fermentation products of the mangrove endophytic fungus *T. flavus* (Li et al., 2011). Of these compounds, compounds **143** and **145** showed cytotoxicity against human cancer cell lines MCF-7 and MDA-MB-435, HepG2, HeLa and PC-3 with IC_50_ values between 0.70 and 2.78 μg/ml. Compound **146**, which was characterized as a new nardosinane-type sesquiterpene and named talaflavuterpenoid A, was isolated from the fermentation products of *T. flavus* (He et al., 2014a).

The new diterpenoid roussoellol C (**147**) was isolated from the fermentation products of *T. purpureogenus* (Wang et al., 2018). Compound **147** had an inhibitory effect on the MCF-7 cancer cell line, with an IC_50_ value of 6.5 μM. A new spiroaxane sesquiterpenoid talaminoid A (**148**) and two drimane sesquiterpenoid talaminoids B and C (**149** and **150**), together with four known compounds (**151**–**154**) were obtained from the fermentation broth of *T. minioluteus* (Nie et al., 2019). Compounds **148**, **151**, and **152** showed significant suppressive effect on the production of NO on LPS-induced BV-2 cells, with IC_50_ values ranging from 4.97 to 7.81 μM. In addition, **148**, **151**, and **152** exhibited significant anti-inflammatory activities against the production of TNF-α and IL-6. Further immunofluorescence experiments revealed the mechanism of action to be inhibitory the NF-kB-activated pathway. The structure of compound **155** was defined and characterized as sordarin, which was isolated from the Australian fungus *Talaromyces* sp. CMB-TU011, which is associated with a marine tunicate (Dewapriya et al., 2017). According to a related study, this compound exhibited antifungal activity (Domínguez et al., 1998). Four new sesquiterpene lactones (**156**–**159**) and three known compounds, purpuride (**151**), berkedrimane B (**152**) and purpuride B (**160**), were isolated from cultures of the marine fungus *T. minioluteus* (Ngokpol et al., 2015). Compounds **152, 156, 159** exhibited weak cytotoxic activity against the HepG2 cancer cell line.

### Meroterpenoids

**Figure 10:**
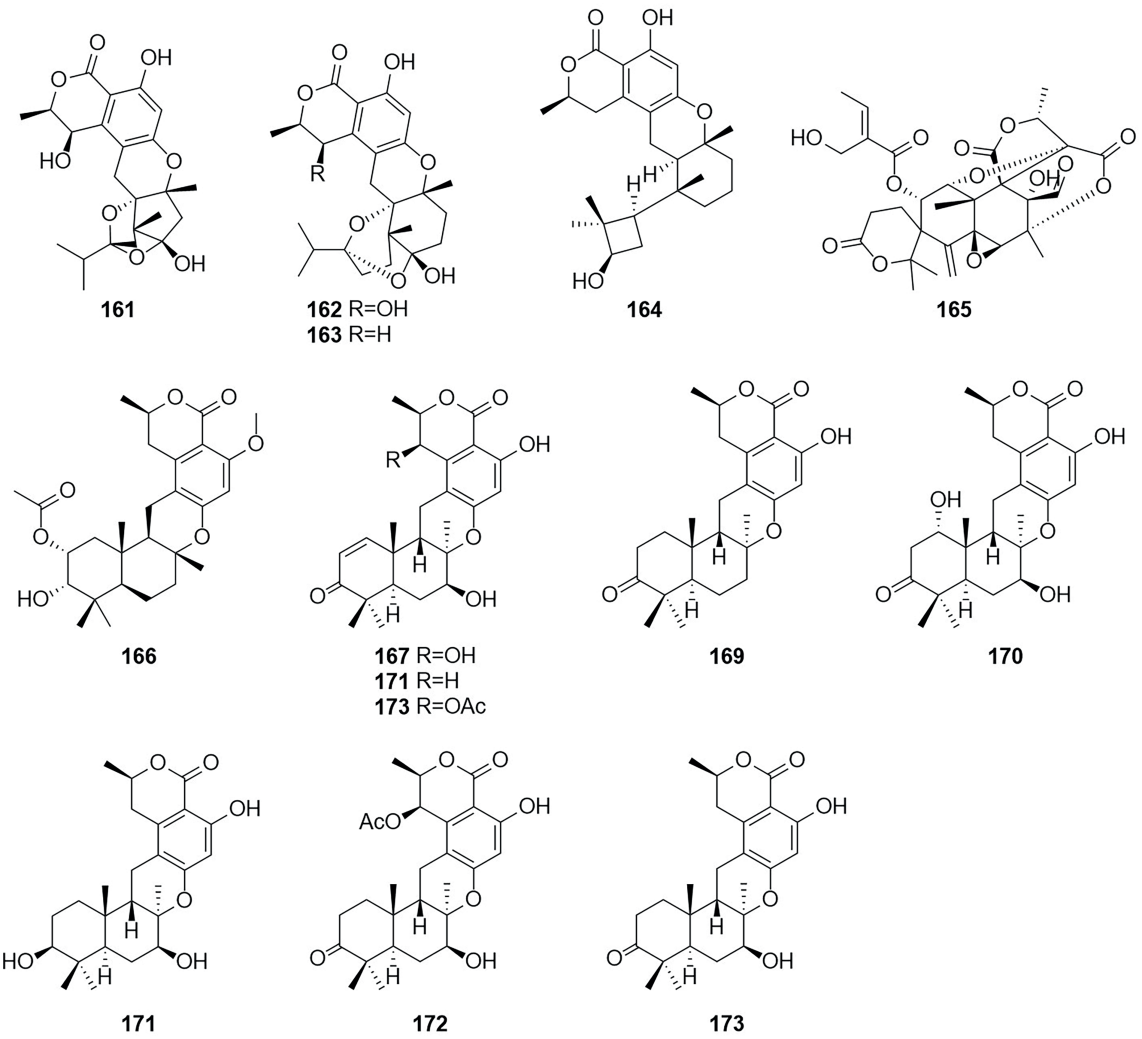
Chemical structures of compounds **161–173**.

Meroterpenoids are natural products that are partially derived from terpenoid biosynthetic pathways, since the prefix “mero-” has the meanings of “part,” “partial,” and “fragment” (Matsuda and Abe, 2020). Four meroterpenoids talaromyolides A–D (**161**–**164**) and Talaromytin (**165**) (Figure 10) were isolated from the marine fungus *Talaromyces* sp. CX11 (Nie et al., 2019). Compound **164** exhibited potent antiviral activity against pseudorabies virus (PRV) with a IC_50_ value of 3.35 μM. Activity tests showed that this compound did not exhibit *in vitro* growth-inhibiting activity against MCF-7 breast adenocarcinoma, NCI-H460 non-small-cell lung cancer, or A375-C5 melanoma cell lines by a method based on the protein-binding dye sulforhodamine B.

A new meroterpenoid, taladrimanin A (**166**), was isolated from the marine-derived fungus *Talaromyces* sp. HM6-1-1. Compound **166** exhibited antitumor activity against MGC803 and MKN28 gastric cancer cells; it also inhibited colony formation and induced apoptosis in MGC803 cells both in a concentration-dependent manner. Additionally, **166** displayed selective antibacterial activity against *S. aureus* 6538P, and low activities toward strains of *V. parahaemolyticus* and *E. coli* (Hong et al., 2022). The structures of compounds **167**–**173**, which were obtained from the fermentation products of the soil fungus *Talaromyces* sp. YO-2 in Osaka, Japan, were defined and characterized as the seven meroterpenoids chrodrimanin A–H (Hayashi et al., 2012a,b). Chrodrimanin B (**168**) exhibited insecticidal activity with an LD_50_ value of 10 ug/g of diet. Chrodrimanins D–F (**170**–**172**) showed insecticidal activity against silkworms with respective LD_50_ values of 20, 10, and 50 ug/g of diet. Compounds 145–148, which were identified as the four meroterpenoid compounds talarolutin A–D, were isolated from the fermentation broth of a strain of the fungus *T. minioluteus* obtained from healthy surface sterilized leaves of milk thistle (Kaur et al., 2016).

### Steroids

**Figure 11:**
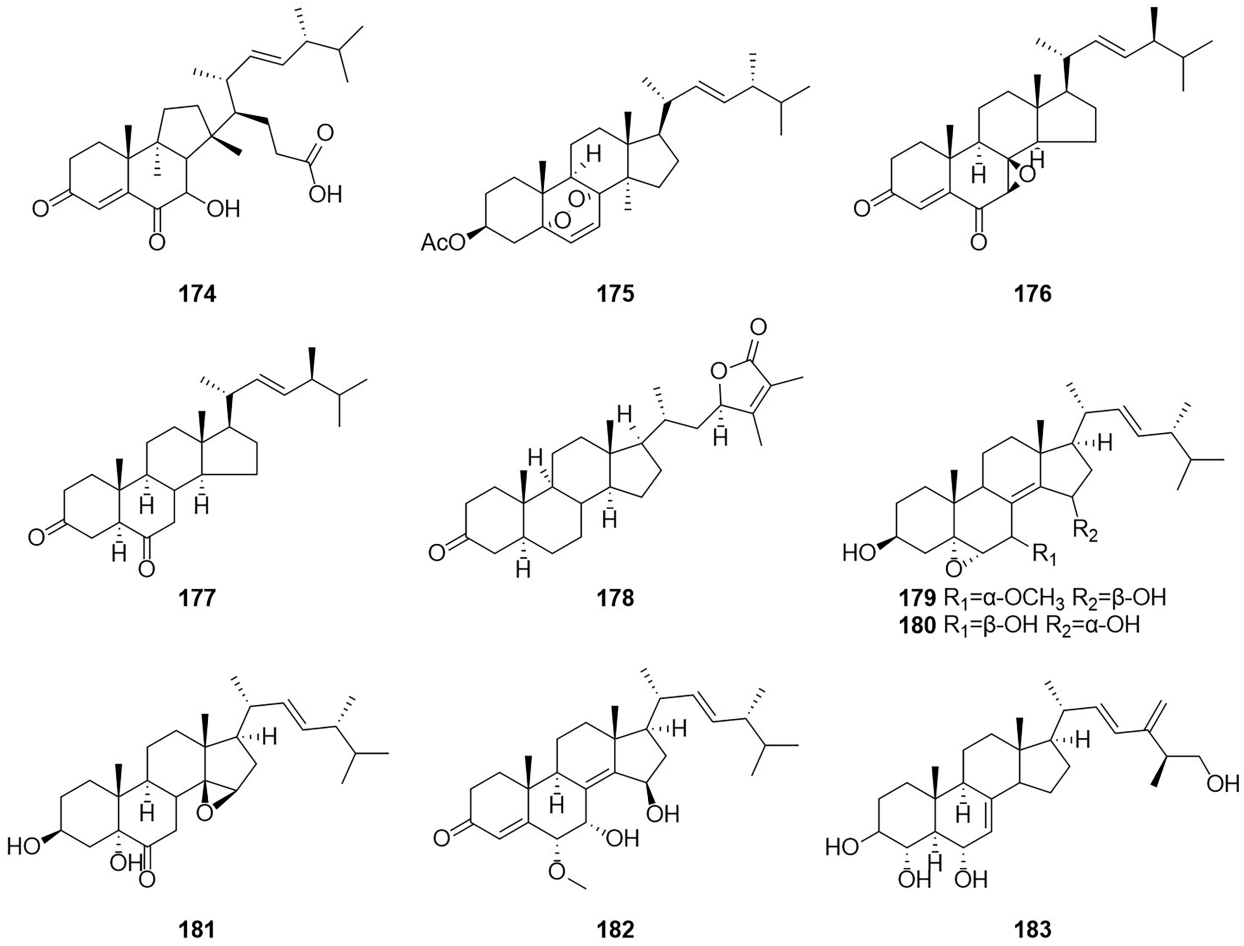
Chemical structures of compounds **174–183**.

Steroids are extremely important medicinally active organic compounds with four rings constructed in a highly specific perhydrocyclopentano[α]phenanthrene orientation. In general, the steroid core structure has 17 carbon atoms connected with 4 fused rings in a specific way. Three of these are cyclohexanes (A, B, and C) and one is cyclopentane system (D ring) (Borah and Banik, 2020). Talasterone A (**174**) (Figure 11), an unprecedented 6/6/5 tricyclic 13 (14 → 8) abeo-8,14-seco-ergostane steroid, was characterized from *T. adpressus* isolated from soil collected from Yalong Bay in Sanya, Hainan (Zhang et al., 2022a). A new compound 3-acetylergosterol-5,8-endoperoxide (**175**) was obtained from the fermentation products of the sponge endophytic fungus *T. trachyspermus* KUFA 0021 (Kuml et al., 2014). In 2017, the new compound talarosterone (**176**) and cyathisterone (**177**) were obtained from the fermentation products of the sponge fungus *T. stipitatus* KUFA 0207 (Noinart et al., 2017). A new withanolide, talasteroid (**178**) was obtained from rice culture of the marine-derived fungus *T. stollii* HBU-115 (Zhang et al., 2022c). Five undescribed sterol derivatives (**179–183**), (22E,24R)-7α-methoxy-5α,6α-epoxyergosta-8(14),22-diene-3β,15β-diol, (22E,24R)-5α,6α-epoxyergosta-8(14),22-diene-3β,7β,15α-triol, (22E,24R)-3β,5α-dihydroxy-14β,15β-epoxyergosta-7,22-diene-6-one, (22E,24R)-6α-methoxy-7α,15β-dihydroxyergosta-4,8(14),22-triene-3-one, and (25S)-ergosta-7,24(28)-diene-3β,4α,6α,26-tetraol were isolated from the extract of *T. stipitatus* (Zhang et al., 2021). The antiproliferative activities of compound **179**–**183** were mainly mediated by inducing cell apoptosis.

### Nitrogen-containing compound

#### Alkaloids

**Figure 12:**
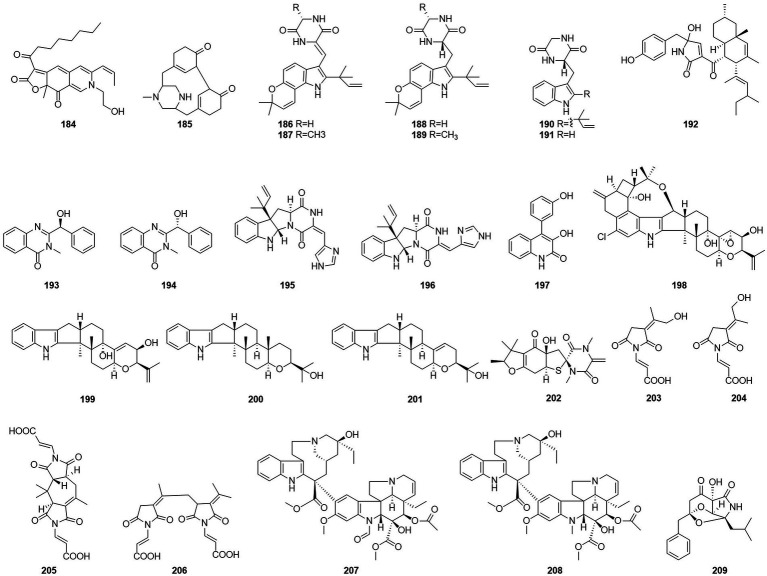
Chemical structures of compounds **184–209**.

Alkaloids are structurally diverse compounds generally classified as such due to the basic character of the molecule (from Latin alkali) and a presence of at least one nitrogen atom, preferably in a heterocycle (Zotchev, 2013). The compound PP-R (**184**) (Figure 12) was isolated from *T. atroroseus* (Frisvad et al., 2013). The red pigments is of interest for the industry as they are stable and non-toxic and can be used as food colorants. Herquline B (**185**) was isolated from the culture filtrate of an endophytic strain of *T. pinophilus* obtained from the strawberry tree (*A. unedo*) (Vinale et al., 2017). In 2011, six indole alkaloids, talathermophilins A–E (**186**–**188**,**190**–**191**) and cyclo(glycyltryptophyl) (**189**), were obtained from the thermophilic fungal strain *T. thermophilus* YM3-4 (Guo et al., 2011, 3–4). ZG-1494α (**192**) was isolated from an ethyl acetate extract of a culture broth of *T. atroroseus* (Frisvad et al., 2013). According to a related study, compound **192** can be used as a novel inhibitor of platelet-activating factor acetyl-transferase (West et al., 1996). Nine alkaloids, 2-[(S)-hydroxy(phenyl)methyl]-3-methylquinazolin-4(3H)-one (**193**), 2-[(R)-hydroxy(phenyl)methyl]-3-methylquinazolin-4(3H)-one (**194**), roquefortine C (**195**), Z-roquefortine C (**196**), viridicatol (**197**), penitrem A (**198**), penijanthine A (**199**), paspaline (**200**), and 3-deoxo-4b-deoxypaxilline (**201**), were isolated from the fermentation broth of the algal endophytic fungus *Talaromyces* sp. *cf*-16 in 2014, of which compounds **196**–**199** could inhibit *S. aureus* (Yang et al., 2016).

Five new compounds, namely talaromanloid A (**202**), 10-hydroxy-8-demethyltalaromydine and 11-hydroxy-8-demethyltalaromydine (**203** and **204**) and ditalaromylectones A and B (**205** and **206**) were identified from the marine-derived fungus *T.* mangshanicus BTBU20211089, which was isolated from a sediment sample collected from the South China Sea. Compound **205** showed an inhibitory effect against *C. albicans* with an MIC value of 200 μg/ml (Zhang et al., 2022b). The endophytic fungus *T. radicus* isolated from Catharanthus roseus was cultured in M2 liquid fermentation medium and PDA fermentation medium. Vincristine (**207**) and vinblastine (**208**) were obtained from this fungus, of which HeLa cells exhibited the highest susceptibility to vincristine. In addition, the apoptosis-inducing activity of vincristine obtained from this fungus was established *via* cell cycle analysis, loss of mitochondrial membrane potential, and DNA fragmentation patterns (Palem et al., 2015). In 2017, the alkaloid talaramide A (**209**) was obtained by culturing of the mangrove endophytic fungus *Talaromyces* sp. HZ-YX1 on a solid rice medium with sea water displayed promising inhibition of the activity of mycobacterial protein kinase G, with an IC_50_ value of 55 μM. A possible biosynthetic pathway was proposed in the paper (Chen et al., 2017).

#### Amides

**Figure 13:**
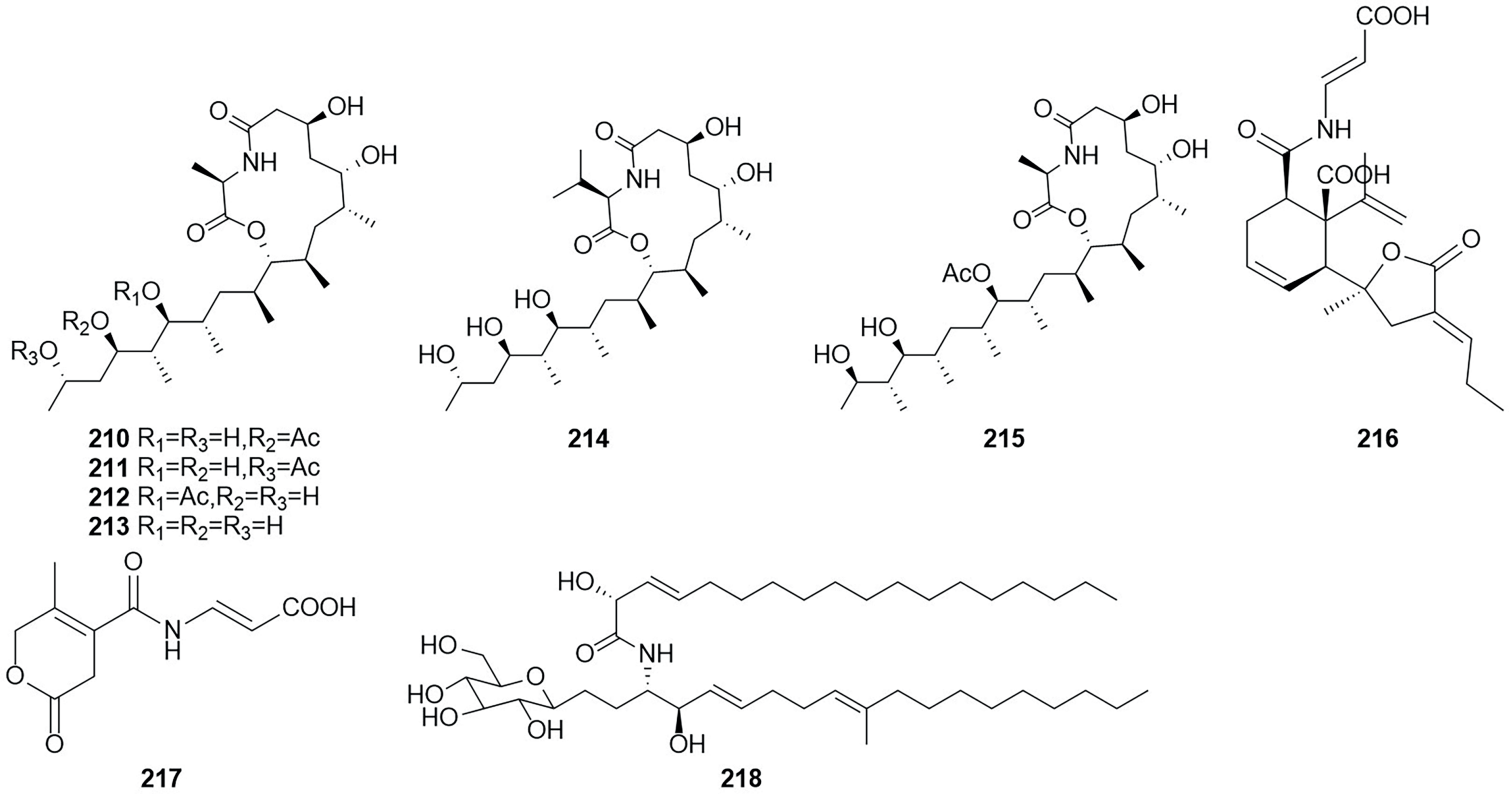
Chemical structures of compounds **210–218**.

Amides are amines with a carbonyl group associated with the ammonia-associated carbon (Jackson, 2008). Six macrolides, thermolides A–F (**210**–**215**) (Figure 13), were isolated from the fermentation products of the thermophilic fungus *T. thermophilus* in 2012 (Guo et al., 2012). Of these compounds, compounds **210** and **211** exhibited strong inhibitory activity against nematodes, with LC_50_ values of 0.5–1.0 μg/ml. Two new compounds, namely talaromydene (**216**) and talaromylectone (**217**) were identified from the marine-derived fungus *T. mangshanicus* BTBU20211089, which was isolated from a sediment sample collected from the South China Sea (Zhang et al., 2022b). Cerebroside C (**218**) was obtained from the endophytic fungus *T. purpureogenus* hosted in *Tylophora ovate* (Zhao et al., 2020).

### Acid

**Figure 14:**
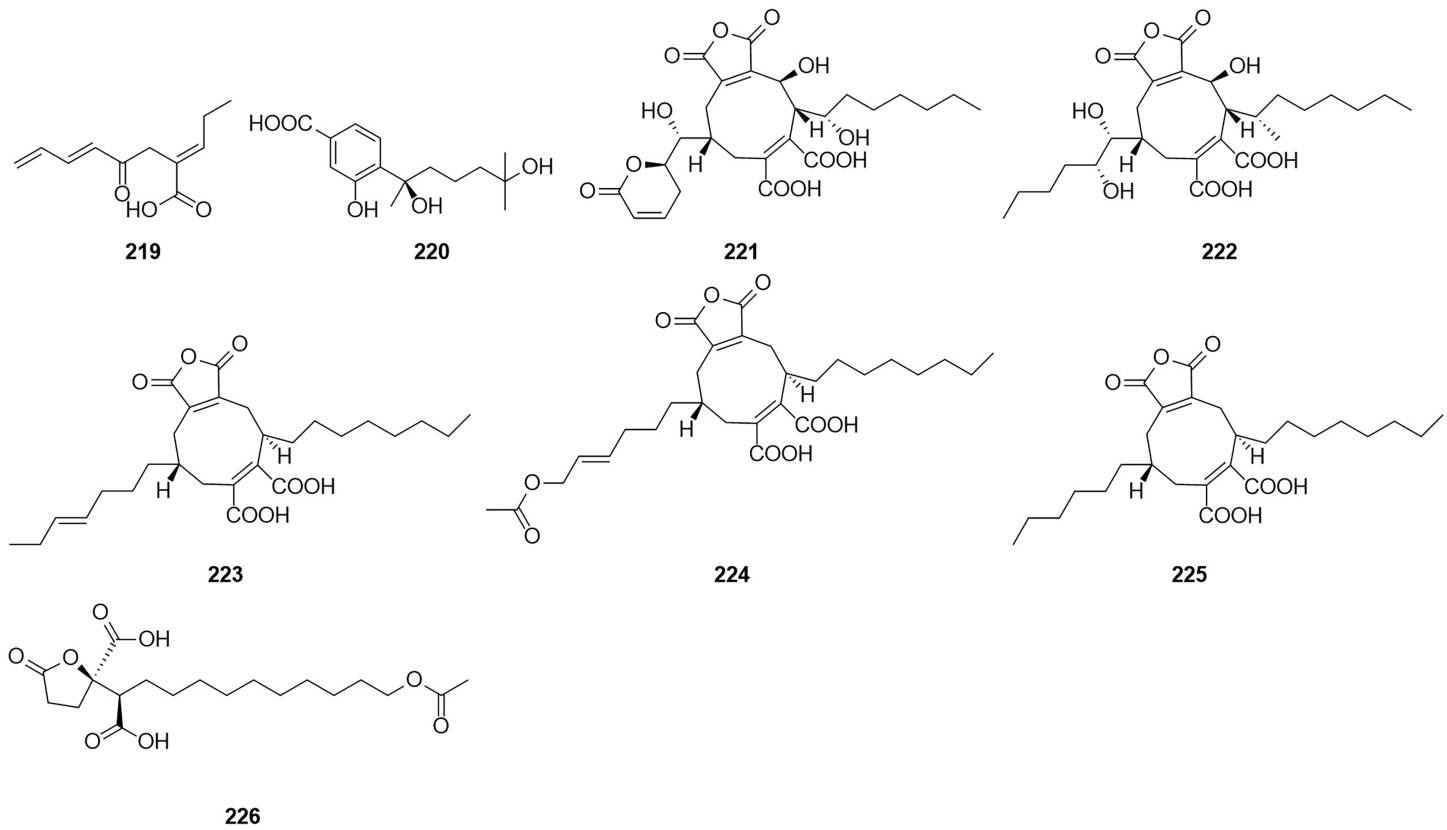
Chemical structures of compounds **219–226**.

A compound, namely, (R)-2-[5-methoxycarbonyl-4-methyl-6-oxo-3,6-dihydro-2H-pyran-2-yl] acetic acid (61), which was obtained from cultures of the endophytic fungus *T. purpureogenus* hosted in *T. ovate*, showed some inhibitory activity against XOD at a concentration of 10 μM with the inhibition rate of 69.9% (Zhao et al., 2020). A new octadienoic acid derivative, oxoberkedienoic acid (**219**) (Figure 14), was isolated from a culture of *T. verruculosus* FKI-5393. The IC_50_ value against Jurkat cells of **219** was 6.1 μg/ml (Sakai et al., 2018). The IC_50_ value against Jurkat cells of **219** was 6.1 mg/ml. (R)-(−)-Hydroxysydonic acid (**220**) was isolated from the strain *Talaromyces* sp. C21-1 obtained from the coral Porites pukoensis collected in Xuwen, Guangdong Province (Nie et al., 2019). The compound **220** showed moderate inhibitory activities to *C. albicans* and methicillin-resistant *S. aureus* (MRSA) with the MICs at 0.075 mM and 0.2 mM, respectively. Rubratoxin acid A-E (**221**–**225**) were isolated from the endophytic fungus *T. purpureogenus* obtained from fresh leaves of the toxic medicinal plant *T. ovate* (Zhao et al., 2019a). Compound **221** showed significant inhibitory activity against NO production in LPS-induced RAW264.7 cells with an IC_50_ value of 1.9 μM. Compounds **222** showed moderate inhibitory activities toward XOD and PTP1b at 10 μM with inhibition rates of 67%. Compound **226**, which was identified as a new spiculisporic acid derivative, spic ulisporic acid E, was isolated from a culture of the fungus *T. trachyspermus* KUFA 0021, which is associated with a marine sponge (Kuml et al., 2014).

### Others

**Figure 15:**
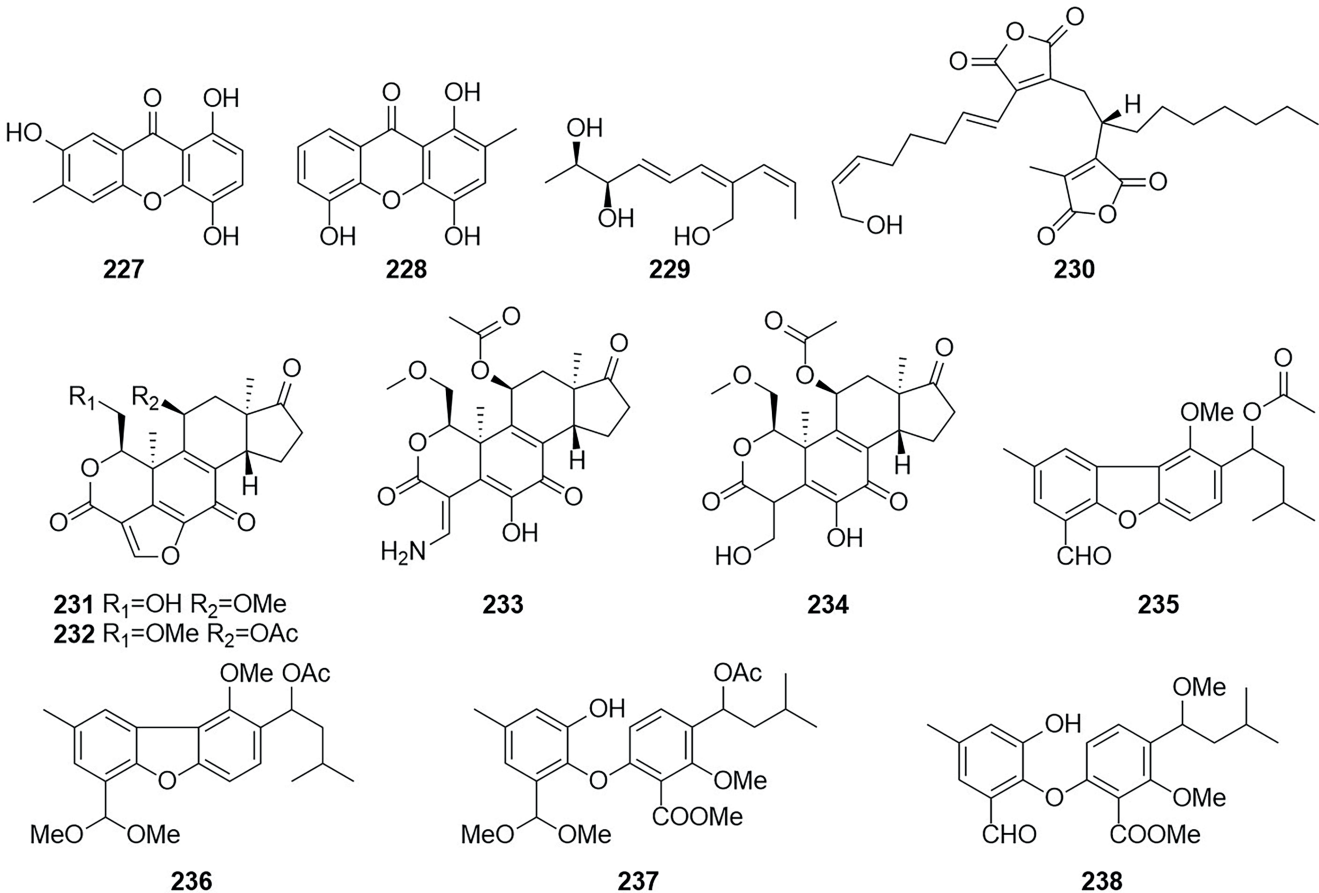
Chemical structures of compounds **227–238**.

The compounds 2,2',3,5'-tetrahydroxy-3'-methylbenzophenone (**227**) and 2,2',5'-trihydroxy-3-methoxy-3'-methylbenzophenone (**228**) (Figure 15), were obtained from *T. islandicus* EN-501, which is an endophytic fungus obtained from the freshly collected marine red alga *Laurencia okamurai* (Li et al., 2016). Compounds **227**–**228** showed strong antioxidant activity against DPPH and ABTS radicals with IC_50_ values of 0.58 ~ 6.92 μg/ml, which were stronger than the positive controls BHT and ascorbic acid. Compounds **227** displayed potent activities against three human pathogens (*E. coli*, *Pseudomonas aeruginosa*, and *S. aureus*) and three aquatic bacteria (*V. alginolyticus*, *V. harveyi*, and *V. parahaemolyticus*) with MIC values ranging from 4 to 32 μg/ml. compound **228** showed weak activity against the tested bacteria (IC_50_ > 64 μg/ml), suggesting that methoxylation at C-3 weakened the antibacterial activities. A new phenylpentenol, wortmannine H (**229**), was isolated from *T. wortmannii* LGT-4, which is an endophytic fungus obtained from *T. wilfordii* (Li et al., 2021).

Talarodride (**230**) were isolated from the endophytic fungus *T. purpureogenus* obtained from fresh leaves of the toxic medicinal plant *T. ovate* (Zhao et al., 2019a). Compounds **230** showed moderate inhibitory activities toward XOD and PTP1b, respectively at 10 μM with inhibition rates of 76%. Four wortmannin derivative compounds, wortmannin B (**231**), wortmannin (**232**), amino adduct 3a (**233**), and wortmannin-diol (VIII) (**234**), were obtained from cultures of the aloe endophytic fungus *T. wortmannii* in 2013 (Bara et al., 2013a) Three new diphenyl ether derivatives, talaromycins A–C (**235**–**237**), together with a known analog (**238**), were obtained from a gorgonian-derived *Talaromyces* sp. (Chen et al., 2015). Compounds **237** showed potent antifouling activities against the larval settlement of the barnacle *Balanus amphitrite* with the EC_50_ values ranging from 2.2 to 4.8 mg/ml. Compounds **238** showed strong cytotoxicity against the human hepatoma HepG2 and Hep3B, human breast cancer MCF-7/ADR, human prostatic cancer PC-3, and human colon carcinoma HCT-116 cell lines with the IC_50_ values ranging from 4.3 to 9.8 mM.

## Summary

Owing to their wide variety of species and abundance in secondary metabolites, *Talaromyces* fungi have great potential in medicine, food, cosmetics, agriculture, and environmental protection. In this paper, the secondary metabolites produced by *Talaromyces* species that have been studied over the past several years are classified and summarized according to the types of compounds (Table 1). Secondary metabolites from more than ten *Talaromyces* species, including *T. wortmannii*, *T. pinophilus*, *T. flavus*, *T. stipitatus*, *T. purpureogenus*, and *T. minioluteus*, have been covered in this paper. These metabolites included 89 esters, 35 polyketones, 16 anthraquinone, 20 terpenoids, 13 meroterpenoids, 10 steroids, 35 nitrogen compounds, 8 acids, and 12 other compounds. Most of these compounds have useful biological activities, such as anti-inflammatory, antibacterial, antitumor, hypolipidemic, or nematocidal activities or inhibition of α-glucosidase, xanthine oxidase, acetyltransferase, NADH fumarate reductase, PI3K-α, Aβ42 aggregation, or the production of NO induced by lipopolysaccharide.

**Table 1:** Name of *Talaromyces’* secondary metabolites, source strains, activity and their culture media.

Category	Compound name	Fungus	Pharmacological activity or application	Medium	References
Esters	Dinapinones AB1 (**1**)	*T. pinophilus* FKI-3864	/	Miura’s medium	Kawaguchi et al., 2013	
	Dinapinones AB2 (**2**)	*T. pinophilus* FKI-3864	Inhabit triacylglycerol synthesis in intact mammalian cells, with an IC_50_ value of 1.17 *μ*M	Miura’s medium	Kawaguchi et al., 2013	
	Dinapinones AC1 (**3**)	*T. pinophilus* FKI-3864	/	Miura’s medium	Kawaguchi et al., 2013	
	Dinapinones AC2 (**4**)	*T. pinophilus* FKI-3864	/	Miura’s medium	Kawaguchi et al., 2013	
	Dinapinones AD1 (**5**)	*T. pinophilus* FKI-3864	/	Miura’s medium	Kawaguchi et al., 2013	
	Dinapinones AD2 (**6**)	*T. pinophilus* FKI-3864	/	Miura’s medium	Kawaguchi et al., 2013	
	Dinapinones AE1 (**7**)	*T. pinophilus* FKI-3864	/	Miura’s medium	Kawaguchi et al., 2013	
	Dinapinones AE2 (**8**)	*T. pinophilus* FKI-3864	/	Miura’s medium	Kawaguchi et al., 2013	
	Talapolyesters A (**9**)	*T. flavus*	/	potato dextrose agar (PDA); potato dextrose broth (PDB); rice solid medium	He et al., 2014b	
	Talapolyesters B (**10**)	*T. flavus*	/	PDA; PDB; rice solid medium	He et al., 2014b	
	Talapolyesters C (**11**)	*T. flavus*	/	PDA; PDB; rice solid medium	He et al., 2014b	
	Talapolyesters D (**12**)	*T. flavus*	/	PDA; PDB; rice solid medium	He et al., 2014b	
	15G256ν (**13**)	*T. flavus*	/	PDA; PDB; rice solid medium	He et al., 2014b	
	15G256ν-me (**14**)	*T. flavus*	/	PDA; PDB; rice solid medium	He et al., 2014b	
	15G256π (**15**)	*T. flavus*	/	PDA; PDB; rice solid medium	He et al., 2014b	
	15G256β-2 (**16**)	*T. flavus*	/	PDA; PDB; rice solid medium	He et al., 2014b	
	15G256α-2 (**17**)	*T. flavus*	/	PDA; PDB; rice solid medium	He et al., 2014b	
	15G256α-2-me (**18**)	*T. flavus*	/	PDA; PDB; rice solid medium	He et al., 2014b	
	15G256ι (**19**)	*T. flavus*	Antitumor	PDA; PDB; rice solid medium	He et al., 2014b	
	15G256β (**20**)	*T. flavus*	Antitumor	PDA; PDB; rice solid medium	He et al., 2014b	
	15G256α (**21**)	*T. flavus*	Antitumor	PDA; PDB; rice solid medium	He et al., 2014b	
	Talapolyesters E (**22**)	*T. flavus*	Antitumor	PDA; PDB; rice solid medium	He et al., 2014b	
	15G256α-1 (**23**)	*T. flavus*	Antitumor	PDA; PDB; rice solid medium	He et al., 2014b	
	Talapolyesters E (**24**)	*T. flavus*	Antitumor	PDA; PDB; rice solid medium	He et al., 2014b	
	15G256ω (**25**)	*T. flavus*	Antitumor	PDA; PDB; rice solid medium	He et al., 2014b	
	Talaromycolides A (**26**)	*T. pinophilus* AF-02	Antibacterial	YES liquid medium	Zhai et al., 2015	
	Talaromycolides B (**27**)	*T. pinophilus* AF-02	Antibacterial	YES liquid medium	Zhai et al., 2015	
	Talaromycolides C (**28**)	*T. pinophilus* AF-02	Antibacterial	YES liquid medium	Zhai et al., 2015	
	Rubralide C (**29**)	*T. pinophilus* AF-02	/	YES liquid medium	Zhai et al., 2015	
	Sclerotinin A (**30**)	*T. pinophilus* AF-02	/	YES liquid medium	Zhai et al., 2015	
	Alternariol (**31**)	*T. pinophilus* AF-02	/	YES liquid medium	Zhai et al., 2015	
	Penicillide (**32**)	*T. pinophilus* AF-02	/	YES liquid medium	Zhai et al., 2015	
	Deacetylisowortmins A (**33**)	*T. wortmannii* LGT-4	/	PDA	Fu et al., 2016	
	Deacetylisowortmins B (**34**)	*T. wortmannii* LGT-4	/	PDA	Fu et al., 2016	
	Talaromyones A (**35**)	*T. stipitatus* SK-4	/	Autoclaved wheat solid-substrate medium	Cai et al., 2017	
	Talaromyones B (**36**)	*T. stipitatus* SK-4	Antibacterial; inhibitα-glucosidase	Autoclaved wheat solid-substrate medium	Cai et al., 2017	
	Purpactin A (**37**)	*T. stipitatus* SK-4	Inhibit α-glucosidase	Autoclaved wheat solid-substrate medium	Cai et al., 2017	
	Butenolides (**38**–**42**)	*T. rugulosus*	/	Solid rice medium	Küppers et al., 2017	
	(3S)-resorcylide derivatives (**43**–**49**)		/			
	Talarodilactones A and B (**50** and **51**)		Antitumor			
	Talaromycin A (**52**)	*Talaromyces* sp. MH551540	Antitumor	Co-culture with *X. angustiphylla*	Yuan et al., 2018	
	Clearanol A (**53**)		Antitumor			
	Wortmannine *F* (**54**)	*T. wortmannii* LGT-4	Antitumor	King’s B Medium	Zhao et al., 2019b	
	Pentalsamonin (**55**)	*T. purpureogenus* CFRM02	Antibacterial	Bengal gram husk (BegH)	Pandit et al., 2018	
	Talaromarnine A (**56**)	*T. marneffei*	/	Corn medium	Yang et al., 2021	
	Talaromarnine B (**57**)	*T. marneffei*	/	Corn medium	Yang et al., 2021	
	Amestolkins A (**58**)	*T. amestolkiae*	Anti-inflammatory	M1 liquid medium	Huang et al., 2022	
	Amestolkins B (**59**)					/		
	Talacoumarins A (**60**)	*T. flavus*	Anti-Aβ42 aggregation activity	PDA; PDB; rice	He et al., 2014c	
	Talacoumarins B (**61**)	*T. flavus*	Anti-Aβ42 aggregation activity	PDA; PDB; rice	He et al., 2014c	
	Chloropestalasin A (**62**)	*T. amestolkiae*	/	Solid cultures	El-Elimat et al., 2021	
	3-Hydroxymethyl-6,8-dimethoxycoumarin (**63**)	*T. amestolkiae*	/	Solid cultures	El-Elimat et al., 2021	
	Pestalasin A (**64**)	*T. amestolkiae*	/	Solid cultures	El-Elimat et al., 2021	
	Dihydroisocoumarins (**65**–**67**)	*T. rugulosus*		Solid rice medium	Küppers et al., 2017	
	Talaromarins A-F (**68**–**73**)	*T. flavus* TGGP35; *Talaromyces* sp. ZZ1616	Antioxidant; antimicrobial	PDB; rice solid medium	Cai et al., 2022; Ma et al., 2022	
	Analogues (**67**,**74**–**89**)	*T. flavus* TGGP35	Antioxidant	Rice solid medium	Cai et al., 2022
Polyketons	Mitorubrin (**90**)	*T. atroroseus*	Red pigment production	Solid medium	Frisvad et al., 2013	
	Monascorubrin (**91**)	*T. atroroseus*	Red pigment production	Solid medium	Frisvad et al., 2013	
	Talaroxanthone (**92**)	*Talaromyces* sp.	/	ISP2-agar medium	Koolen et al., 2013	
	9a-Epi-bacillisporin E (**93**)	*T. stipitatus*	/	PDA	Zang et al., 2016	
	1-Epi-bacillisporin *F* (**94**)	*T. stipitatus*	/	PDA	Zang et al., 2016	
	Bacillisporins F-H (**95**–**97**)	*T. stipitatus*	Antibacterial	PDA	Zang et al., 2016	
	Wortmannilactones I1-I3(98–100)	*T. wortmannii*	Antioxidant	Corn plate medium	Liu et al., 2016	
	Talaraculones A–F (**102**–**107**)	*T. aculeatus*	Inhibit α-glucosidase	PDA	Ren et al., 2017	
	Pinazaphilone B (**108**)	*T. aculeatus*	Inhibit α-glucosidase	PDA	Ren et al., 2017	
	Pinophilin B (**109**)	*T. aculeatus*	/	PDA	Ren et al., 2017	
	Sch 725680 (**110**)	*T. aculeatus*	/	PDA	Ren et al., 2017	
	(−)-Mitorubrin (**111**)	*T. aculeatus*	/	PDA	Ren et al., 2017	
	(−)-Mitorubrinol (**112**)	*T. aculeatus*	/	PDA	Ren et al., 2017	
	Paecillin D (**113**)	*T. stipitatus*	Antifungal	International streptomyces project 2 liquid medium (ISP2)	da Silva et al., 2017	
	Secalonic acid A (**114**)	*T. stipitatus*	Antifungal	ISP2	da Silva et al., 2017	
	Blennolide G (**115**)	*T. stipitatus*	Antifungal	ISP2	da Silva et al., 2017	
	Versixanthone A (**116**)	*T. stipitatus*	Antifungal	ISP2	da Silva et al., 2017	
	Penicillixanthone A (**117**)	*T. stipitatus*	/	ISP2	da Silva et al., 2017	
	Paecillin B (**118**)	*T. stipitatus*	/	ISP2	da Silva et al., 2017	
	Talarodrides A − F (**119–124**)	*Talaromyces* sp. HDN1820200	Antimicrobial	PDB	Zhao et al., 2021b
Anthraquinone	Skyrin (**125**)	*Talaromyces* sp. ZH-154	Antitumor	PDA, PDB	Liu et al., 2010; Xie et al., 2016	
	Emodin (**126**)	*Talaromyces* sp. ZH-154	Antitumor	PDA, PDB	Liu et al., 2010	
	Biemodin (**127**)	*T. wortmannii*	/	Rice solid medium	Bara et al., 2013a	
	Emodic acid (**128**)	*T. wortmannii*	/	Rice solid medium	Bara et al., 2013a	
	Oxyskyrin (**129**)	*T. wortmannii*	Antitumor	Rice solid medium	Bara et al., 2013a; Xie et al., 2016	
	Rugulosins A - B (**130**–**131**)	*T. wortmannii*	/	Rice solid medium	Bara et al., 2013a	
	Talaromannins A-B (**132**–**133**)	*T. wortmannii*	Antibacterial	Rice solid medium	Bara et al., 2013b	
	3-Demethyl-3-(2-hydroxypropyl)-skyrin (**134**)	*Talaromyces* sp. YE 3016	Antitumor	Rice solid medium	Xie et al., 2016	
	1,3,6-Trihydroxy-8-methylanthraquinone (**135**)	*Talaromyces* sp. YE 3016	/	Rice solid medium	Xie et al., 2016	
	2,2'-bis-(7-methyl-1,4,5-trihydroxy-anthracene-9,10-dione) (**136**)	*T. stipitatus* KUFA 0207	/	Rice solid medium	Noinart et al., 2017	
	Questinol (**137**)	*T. stipitatus* KUFA 0207	Anti-obesity activity	Rice solid medium	Noinart et al., 2017	
	Citreorosein (**138**)	*T. stipitatus* KUFA 0207	Anti-obesity activity	Rice solid medium	Noinart et al., 2017	
	Fallacinol (**139**)	*T. stipitatus* KUFA 0207	/	Rice solid medium	Noinart et al., 2017	
	Rheoemodin (**140**)	*T. stipitatus* KUFA 0207	/	Rice solid medium	Noinart et al., 2017
Terpenoids	Pinophicin A (**141**)	*T. pinophilus*	/	MEB medium	Zhao et al., 2021a	
	Talaperoxides A–D (**142**–**145**)	*T. flavus*	Antitumor	Autoclaved rice solid-substrate medium	Li et al., 2011	
	Talaflavuterpenoid A (**146**)	*T. flavus*	/	Rice solid medium	He et al., 2014a	
	Roussoellol C (**147**)	*T. purpureogenus*	Antitumor	Rice solid medium	Wang et al., 2018	
	Talaminoid A (**148**)	*T. minioluteus*	Anti-inflammatory	Rice solid medium	Chen et al., 2019	
	Talaminoids B - C (**149**–**150**)	*T. minioluteus*	/	Rice solid medium	Chen et al., 2019	
	Purpuride (**151**)	*T. minioluteus*	Anti-inflammatory	Rice solid medium	Chen et al., 2019	
	Berkedrimanes B (**152**)	*T. minioluteus*	Anti-inflammatory	Rice solid medium	Chen et al., 2019	
	Minioluteumide B (**153**)	*T. minioluteus*	/	Rice solid medium	Chen et al., 2019	
	1αHydroxyconfertifolin (**154**)	*T. minioluteus*	/	Rice solid medium	Chen et al., 2019	
	Sordarin (**155**)	*Talaromyces* sp. (CMB-TU011)	Antifungal	M1 agar plate	Domínguez et al., 1998; Dewapriya et al., 2017	
	Four new sesquiterpene lactones (**156**–**159**)	*T. minioluteus*	Antitumor	PDB	Ngokpol et al., 2015	
	Purpuride B (**160**)	*T. minioluteus*	/	PDB	Ngokpol et al., 2015
Meroterpenoid	Talaromyolides A–D (**161**–**164**)	*Talaromyces* sp. CX11	Antiviral	Liquid Medium	Cao et al., 2019	
	Talaromytin (**165**)	*Talaromyces* sp. CX11	/	Liquid Medium	Cao et al., 2019	
	Taladrimanin A (**166**)	*Talaromyces* sp. HM6-1–1	Antitumor activity; antibacterial activity	Rice solid medium	Hong et al., 2022	
	Chrodrimanins A-H (**167**–**173**)	*Talaromyces* sp. YO-2	Antimalarial	Okara	Hayashi et al., 2012a,b
Steroids	Talasterone A (**174**)	*T. adpressus*	Anti-inflammatory	Rice solid medium	Zhang et al., 2022a	
	3-Acetylergosterol-5,8-endoperoxide (**175**)	*Talaromyces trachyspermus* KUFA 0021	/	GPMY	Kuml et al., 2014	
	Talarosterone (**176**)	*T. stipitatus* KUFA 0207	/	Rice solid medium	Noinart et al., 2017	
	Cyathisterone (**177**)		/			
	Talasteroid (**178**)	*T. stollii*	/	PDA	Zhang et al., 2022c	
	(22E,24R)-7α-Methoxy-5α,6α-epoxyergosta-8(14),22-diene-3β,15β-diol (**179**)	*T. stipitatus*	Antiproliferative	Rice solid medium	Zhang et al., 2021	
	(22E,24R)-5α,6α-Epoxyergosta-8(14),22-diene-3β,7β,15α-triol (**180**)	*T. stipitatus*	/	Rice solid medium	Zhang et al., 2021	
	(22E,24R)-3β,5α-Dihydroxy-14β,15β-epoxyergosta-7,22-diene-6-one (**181**)	*T. stipitatus*	/	Rice solid medium	Zhang et al., 2021	
	(22E,24R)-6α-Methoxy-7α,15β-dihydroxyergosta-4,8(14),22-triene-3-one (**182**)	*T. stipitatus*	/	Rice solid medium	Zhang et al., 2021	
	(25S)- Ergosta-7,24(28)-diene-3β,4α,6α,26-tetraol (**183**)	*T. stipitatus*	Antiproliferative	Rice solid medium	Zhang et al., 2021
Alkaloids	PP-R (**184**)	*T. atroroseus*	Food colorants	Solid medium	Frisvad et al., 2013	
	Herquline B (**185**)	*T. pinophilus*	/	Solid medium	Vinale et al., 2017	
	Talathermophilins A–E (**186**–**188**,**190**–**191**)	*T. thermophilus* YM3-4	/	PDB	Guo et al., 2011	
	Cyclo(glycyltryptophyl) (**189**)	*T. thermophilus* YM3-4	/	PDB	Guo et al., 2011	
	ZG-1494α (**192**)	*T. atroroseus*	A novel inhibitor of platelet-activating factor acetyl-transferase	PDB	Frisvad et al., 2013	
	2-[(S)-Hydroxy(phenyl)methyl]-3-methylquinazolin-4(3H)-one (**193**)	*Talaromyces* sp. *cf*-16	/	PDA	Yang et al., 2016	
	2-[(R)-Hydroxy(phenyl)methyl]-3-methylquinazolin-4(3H)-one (**194**)	*Talaromyces* sp. *cf*-16	/	PDA	Yang et al., 2016	
	Roquefortine C (**195**)	*Talaromyces* sp. *cf*-16	/	PDA	Yang et al., 2016	
	Z-Roquefortine C (**196**)	*Talaromyces* sp. *cf*-16	Antibacterial	PDA	Yang et al., 2016	
	Viridicatol (**197**)	*Talaromyces* sp. *cf*-16	Antibacterial	PDA	Yang et al., 2016	
	Penitrem A (**198**)	*Talaromyces* sp. *cf*-16	Antibacterial	PDA	Yang et al., 2016	
	Penijanthine A (**199**)	*Talaromyces* sp. *cf*-16	Antibacterial	PDA	Yang et al., 2016	
	Paspaline (**200**)	*Talaromyces* sp. *cf*-16	/	PDA	Yang et al., 2016	
	3-Deoxo-4b-deoxypaxilline (**201**)	*Talaromyces* sp. *cf*-16	/	PDA	Yang et al., 2016	
	Talaromanloid A (**202**)	*T. mangshanicus* BTBU20211089	/	Rice solid medium	Zhang et al., 2022b	
	10-Hydroxy-8-demethyltalaromydine (**203**)	*T. mangshanicus* BTBU20211089	/	Rice solid medium	Zhang et al., 2022b	
	11-Hydroxy-8-demethyltalaromydine (**204**)	*T. mangshanicus* BTBU20211089	/	Rice solid medium	Zhang et al., 2022b	
	Ditalaromylectones A (**205**)	*T. mangshanicus* BTBU20211089	Antibacterial	Rice solid medium	Zhang et al., 2022b	
	Ditalaromylectones A (**206**)	*T. mangshanicus* BTBU20211089	/	Rice solid medium	Zhang et al., 2022b	
	Vincristine (**207**)	*T. radicus*	Antitumor	M2 liquid medium; PDA	Palem et al., 2015	
	Vinblastine (**208**)		/			
	Talaramide A (**209**)	*Talaromyces* sp. HZ-YX1	Antibacterial	Solid rice medium	Chen et al., 2017
Amides	Thermolides A–F (**210**–**215**)	*T. thermophilus*	**210**–**211**: Insect resistance	PDA	Guo et al., 2012	
	Talaromydene (**216**)	*T. mangshanicus* BTBU20211089	/	Rice solid medium	Zhang et al., 2022b	
	Talaromylectone (**217**)	*T. mangshanicus* BTBU20211089	/	Rice solid medium	Zhang et al., 2022b	
	Cerebroside C (**218**)	*T. purpurogenus*	/		Zhao et al., 2020
Acid	Oxoberkedienoic acid (**219**)	*T. verruculosus* FKI-5393	Antitumor	Rice solid medium	Sakai et al., 2018	
	(R)-(−)-Hydroxysydonic acid (**220**)	*Talaromyces* sp. C21-1	Antimicrobial	Liquid medium	Nie et al., 2019	
	Rubratoxin acid A-E (**221**–**225**)	*T. purpureogenus*	**221**: Anti-inflammatory **222**: Antioxidant	PDA	Zhao et al., 2019b	
	Spic ulisporic acid E (**226**)	*T. trachyspermus* KUFA 0021	/	GPMY	Kuml et al., 2014
Others	2,2',3,5'-tetrahydroxy-3'-methylbenzophenone (**227**)	*T. islandicus* EN-501	Antioxidant; antibacterial activity	Rice solid medium	Li et al., 2016	
	2,2',5'-trihydroxy-3-methoxy-3'-methylbenzophenone (**228**)	*T. islandicus* EN-501	Antioxidant; antibacterial Activity	Rice solid medium	Li et al., 2016	
	Wortmannine H (**229**)	*T. wortmannii* LGT-4	/	Martin medium	Li et al., 2021	
	Talarodride (**230**)	*T. purpurogenus*	Antitumor	Rice solid medium	Zhao et al., 2019b	
	Wortmannin B (**231**)	*T. wortmannii*	/	Rice solid medium	Bara et al., 2013a	
	Wortmannin (**232**)	*T. wortmannii*	/	Rice solid medium	Bara et al., 2013a	
	Amino adduct 3a (**233**)	*T. wortmannii*	/	Rice solid medium	Bara et al., 2013a	
	Wortmannin-diol (VIII) (**234**)	*T. wortmannii*	/	Rice solid medium	Bara et al., 2013a	
	Talaromycins A–C (**235**–**237**)	*Talaromyces* sp. SBE-14 (EU236708) Talaromyces sp. SBE-14 (EU236708)	Antifouling	PDA	Chen et al., 2015	
	Tienilic acid A methyl ester (**238**)		/	PDA	Chen et al., 2015

## Prospects

*Talaromyces* fungi include some of the most important species of microorganisms. The secondary metabolites from *Talaromyces* species that have unique structures and useful activities are of great value in research and development. However, there are still some problems to be solved in the study of fungal secondary metabolites. Firstly, owing to the limitations of strain isolation techniques and fungal culture conditions, some fungi cannot be isolated or do not grow well. Now often use fungal culture mediums are: PDA medium, PDB medium, BegH medium, rice solid medium and so on. Among them, rice medium is the most used (Table 1), which may be due to more fungal metabolites cultured in solid medium than liquid medium. It also reflected the problems of single nutrition and limited culture in the application of fungus synthesis medium. It is hoped that unconventional media can be used and new media can be developed. Secondly, it had been reported in available reports that the addition of epigenetic modifications to the culture medium can stimulate the expression of silenced genes thereby enabling the production of novel secondary metabolites. However, none of the literature in the study of secondary metabolites of the *Talaromyces* has investigated the effect of epigenetic modifiers. Therefore, epigenetic modifiers can be added to stimulate the expression of their silent genes. Finally, many existing studies have been done on the ethyl acetate part of the ferment, which is moderately polar and easy to separate. The aqueous part, on the other hand, has been ignored or even discarded due to its high polarity and difficulty of separation. Therefore, it is hoped that methods for the separation of compounds with high polarity will be developed as well as the development of related fillers. In a word, we should make full use of modern scientific and technological methods to carry out an in-depth study of the secondary metabolites produced by *Talaromyces* fungi and identify new active components to provide lead compounds for the research and development of innovative drugs.

## Author contributions

L-RL, L-QG, and M-YJ wrote the paper. JG, RW, and RL cultured and identified the fungus. L-RL, M-DL, and LH collected the STM data. YD checked the paper. G-ZW and DW verified the content. All authors have read and agreed to the published version of the manuscript.

## Funding

This research was funded by the National Natural Science Foundation of China (81973189, 81973460), Science and Technology Department of Sichuan Province (2021ZYD0079), Chengdu University of Traditional Chinese Medicine (CZYJC1905, 2020XSGG016, 2020JCRC006, SKL2021-19, SKL2021-42), and National Interdisciplinary Innovation Team of Traditional Chinese Medicine (ZYYCXTD-D-202209).

## Conflict of interest

The authors declare that the research was conducted in the absence of any commercial or financial relationships that could be construed as a potential conflict of interest.

## Publisher’s note

All claims expressed in this article are solely those of the authors and do not necessarily represent those of their affiliated organizations, or those of the publisher, the editors and the reviewers. Any product that may be evaluated in this article, or claim that may be made by its manufacturer, is not guaranteed or endorsed by the publisher.

## References

[ref1] Adefegha S. A. Oboh G. Oluokun O. O. (2022). Chapter 11 – Food bioactives: the food image behind the curtain of health promotion and prevention against several degenerative diseases. Stud. Nat. Prod. Chem. 72, 391–421. doi: 10.1016/B978-0-12-823944-5.00012-0

[ref2] Bara R. Aly A. H. Pretsch A. Wray V. Wang B. Proksch P.. (2013a). Antibiotically active metabolites from *Talaromyces wortmannii*, an endophyte of *Aloe vera*. J. Antibiot. 66, 491–493. doi: 10.1038/ja.2013.28, PMID: 23677029

[ref3] Bara R. Zerfass I. Aly A. H. Goldbach-Gecke H. Raghavan V. Sass P.. (2013b). Atropisomeric dihydroanthracenones as inhibitors of multiresistant *Staphylococcus aureus*. J. Med. Chem. 56, 3257–3272. doi: 10.1021/jm301816a, PMID: 23534483

[ref4] Batista Â. G. Silva-Maia Da J. K. Maróstica M. R. (2021). “Generation and alterations of bioactive organosulfur and phenolic compounds,” in Chemical Changes during Processing and Storage of Foods. eds. Rodriguez-Amaya D. B. Amaya-Farfan J. (London, UK: Elsevier). 537–577.

[ref5] Borah P. Banik B. K. (2020). “12 – Diverse synthesis of medicinally active steroids,” in Green Approaches in Medicinal Chemistry for Sustainable Drug Design Advances in Green and Sustainable Chemistry. ed. Banik B. K. (London, UK: Elsevier), 449–490.

[ref6] Braca A. Bader A. De Tommasi N. (2012). Plant and fungi 3,4-dihydroisocoumarins. Stud. Nat. Prod. Chem 37, 191–215. doi: 10.1016/B978-0-444-59514-0.00007-9

[ref7] Cai R. Chen S. Long Y. Li C. Huang X. She Z. (2017). Depsidones from *Talaromyces stipitatus* SK-4, an endophytic fungus of the mangrove plant *Acanthus ilicifolius*. Phytochem. Lett. 20, 196–199. doi: 10.1016/j.phytol.2017.04.023

[ref8] Cai J. Zhu X. C. Zeng W. N. Wang B. Luo Y. P. Liu J.. (2022). Talaromarins A–F: six new isocoumarins from mangrove-derived fungus *Talaromyces flavus* TGGP35. Mar. Drugs 20, 361. doi: 10.3390/md20060361, PMID: 35736164 PMC9229493

[ref9] Cao X. Shi Y. Wu X. Wang K. Huang S. Sun H.. (2019). Talaromyolides A-D and talaromytin: polycyclic meroterpenoids from the fungus *Talaromyces* sp. CX11. Org. Lett. 21, 6539–6542. doi: 10.1021/acs.orglett.9b02466, PMID: 31364857

[ref10] Chen M. Han L. Shao C. L. She Z. G. Wang C. Y. (2015). Bioactive Diphenyl ether derivatives from a gorgonian-derived fungus *Talaromyces* sp. Chem. Biodivers. 12, 443–450. doi: 10.1002/cbdv.201400267, PMID: 25766917

[ref11] Chen S. He L. Chen D. Cai R. Long Y. Lu Y.. (2017). Talaramide A, an unusual alkaloid from the mangrove endophytic fungus *Talaromyces* sp. (HZ-YX1) as an inhibitor of mycobacterial PknG. New J. Chem. 41, 4273–4276. doi: 10.1039/C7NJ00059F

[ref12] Chen C. Sun W. Liu X. Wei M. Liang Y. Wang J.. (2019). Anti-inflammatory spiroaxane and drimane sesquiterpenoids from *Talaromyces minioluteus* (*Penicillium minioluteum*). Bioorg. Chem. 91:103166. doi: 10.1016/j.bioorg.2019.103166, PMID: 31404796

[ref13] da Silva P. de Souza M. Bianco E. da Silva S. Soares L. Costa E.. (2017). Antifungal polyketides and other compounds from amazonian endophytic *Talaromyces* fungi. J. Braz. Chem. Soc. 29, 622–630. doi: 10.21577/0103-5053.20170176

[ref14] De Stefano S. Nicoletti R. Milone A. Zambardino S. (1999). 3-o-Methylfunicone, a fungitoxic metabolite produced by the fungus *Penicillium pinophilum*. Phytochemistry 52, 1399–1401. doi: 10.1016/S0031-9422(99)00320-9

[ref15] Dethoup T. Kaewsalong N. Songkumorn P. Jantasorn A. (2018). Potential application of a marine-derived fungus, *Talaromyces tratensis* KUFA 0091 against rice diseases. Biol. Control 119, 1–6. doi: 10.1016/j.biocontrol.2017.11.008

[ref16] Dewapriya P. Khalil Z. G. Prasad P. Salim A. A. Cruz-Morales P. Marcellin E.. (2018). Talaropeptides A-D: structure and biosynthesis of extensively N-methylated linear peptides From an Australian marine tunicate-derived *Talaromyces* sp. Front. Chem. 6:394. doi: 10.3389/fchem.2018.00394, PMID: 30234104 PMC6131563

[ref17] Dewapriya P. Prasad P. Damodar R. Salim A. A. Capon R. J. (2017). Talarolide A, a cyclic heptapeptide hydroxamate from an Australian marine tunicate-associated fungus, *Talaromyces* sp. (CMB-TU011). Org. Lett. 19, 2046–2049. doi: 10.1021/acs.orglett.7b00638, PMID: 28383269

[ref18] Domínguez J. M. Kelly V. A. Kinsman O. S. Marriott M. S. Gómez de las Heras F. Martín J. J. (1998). Sordarins: a new class of antifungals with selective inhibition of the protein synthesis elongation cycle in yeasts. Antimicrob. Agents Chemother. 42, 2274–2278. doi: 10.1128/AAC.42.9.2274, PMID: 9736548 PMC105812

[ref19] El-Elimat T. Figueroa M. Raja H. A. Alnabulsi S. M. Oberlies N. H. (2021). Coumarins, dihydroisocoumarins, a dibenzo-α-pyrone, a meroterpenoid, and a merodrimane from *Talaromyces amestolkiae*. Tetrahedron Lett. 72:153067. doi: 10.1016/j.tetlet.2021.153067, PMID: 34421136 PMC8378670

[ref20] Frisvad J. C. Yilmaz N. Thrane U. Rasmussen K. B. Houbraken J. Samson R. A. (2013). *Talaromyces atroroseus*, a new species efficiently producing industrially relevant red pigments. PLoS One 8:e84102. doi: 10.1371/journal.pone.0084102, PMID: 24367630 PMC3868618

[ref21] Fu G.-C. Yang Z.-D. Zhou S.-Y. Yu H.-T. Zhang F. Yao X.-J. (2016). Two new compounds, deacetylisowortmins A and B, isolated from an endophytic fungus, *Talaromyces wortmannii* LGT-4. Nat. Prod. Res. 30, 1623–1627. doi: 10.1080/14786419.2015.1129329, PMID: 26729481

[ref22] Guo J.-P. Tan J.-L. Wang Y.-L. Wu H.-Y. Zhang C.-P. Niu X.-M.. (2011). Isolation of talathermophilins from the thermophilic fungus *Talaromyces thermophilus* YM3-4. J. Nat. Prod. 74, 2278–2281. doi: 10.1021/np200365z, PMID: 21967034

[ref23] Guo Y. Tu T. Yuan P. Wang Y. Ren Y. Yao B.. (2019). High-level expression and characterization of a novel aspartic protease from *Talaromyces leycettanus* JCM12802 and its potential application in juice clarification. Food Chem. 281, 197–203. doi: 10.1016/j.foodchem.2018.12.096, PMID: 30658748

[ref24] Guo J.-P. Zhu C.-Y. Zhang C.-P. Chu Y.-S. Wang Y.-L. Zhang J.-X.. (2012). Thermolides, potent nematocidal PKS-NRPS hybrid metabolites from thermophilic fungus *Talaromyces thermophilus*. J. Am. Chem. Soc. 134, 20306–20309. doi: 10.1021/ja3104044, PMID: 23210772

[ref25] Halo B. A. Al-Yahyai R. A. Maharachchikumbura S. S. N. Al-Sadi A. M. (2019). *Talaromyces variabilis* interferes with pythium aphanidermatum growth and suppresses pythium-induced damping-off of cucumbers and tomatoes. Sci. Rep. 9, 11255. doi: 10.1038/s41598-019-47736-x, PMID: 31375723 PMC6677756

[ref26] Hayashi H. Oka Y. Kai K. Akiyama K. (2012a). A new meroterpenoid, chrodrimanin C, from YO-2 of *Talaromyces* sp. Biosci. Biotechnol. Biochem. 76, 745–748. doi: 10.1271/bbb.110858, PMID: 22484942

[ref27] Hayashi H. Oka Y. Kai K. Akiyama K. (2012b). New chrodrimanin congeners, chrodrimanins D–H, from YO-2 of *Talaromyces* sp. Biosci. Biotechnol. Biochem. 76, 1765–1768. doi: 10.1271/bbb.120365, PMID: 22972343

[ref28] He J.-W. Liang H.-X. Gao H. Kuang R.-Q. Chen G.-D. Hu D.. (2014a). Talaflavuterpenoid A, a new nardosinane-type sesquiterpene from *Talaromyces flavus*. J. Asian Nat. Prod. Res. 16, 1029–1034. doi: 10.1080/10286020.2014.933812, PMID: 25082104

[ref29] He J.-W. Mu Z.-Q. Gao H. Chen G.-D. Zhao Q. Hu D.. (2014b). New polyesters from *Talaromyces flavus*. Tetrahedron 70, 4425–4430. doi: 10.1016/j.tet.2014.02.060

[ref30] He J.-W. Qin D.-P. Gao H. Kuang R.-Q. Yu Y. Liu X.-Z.. (2014c). Two new coumarins from *Talaromyces flavus*. Molecules 19, 20880–20887. doi: 10.3390/molecules191220880, PMID: 25514227 PMC6271517

[ref31] Hong X. Guan X. Lai Q. Yu D. Chen Z. Fu X.. (2022). Characterization of a bioactive meroterpenoid isolated from the marine-derived fungus *Talaromyces* sp. Appl. Microbiol. Biotechnol. 106, 2927–2935. doi: 10.1007/s00253-022-11914-1, PMID: 35416486

[ref32] Huang L.-J. Li X.-A. Jin M.-Y. Guo W.-X. Lei L.-R. Liu R.. (2022). Two previously undescribed phthalides from *Talaromyces amestolkiae*, a symbiotic fungus of *Syngnathus acus*. J. Asian Nat. Prod. Res. 1–9. doi: 10.1080/10286020.2022.2075738, PMID: 35582859

[ref33] Jackson R. S. (2008). Chemical constituents of grapes and wine. Wine Sci. 270–331. doi: 10.1016/B978-012373646-8.50009-3

[ref34] Kaur A. Raja H. A. Swenson D. C. Agarwal R. Deep G. Falkinham J. O.. (2016). Talarolutins A-D: meroterpenoids from an endophytic fungal isolate of *Talaromyces minioluteus*. Phytochemistry 126, 4–10. doi: 10.1016/j.phytochem.2016.03.013, PMID: 27048854 PMC4861051

[ref35] Kawaguchi M. Uchida R. Ohte S. Miyachi N. Kobayashi K. Sato N.. (2013). New dinapinone derivatives, potent inhibitors of triacylglycerol synthesis in mammalian cells, produced by *Talaromyces pinophilus* FKI-3864. J. Antibiot. 66, 179–189. doi: 10.1038/ja.2012.127, PMID: 23532022

[ref36] Koolen H. H. F. Menezes L. S. Souza M. P. Silva F. M. A. Almeida F. G. O. de Souza A. Q. L.. (2013). Talaroxanthone, a novel xanthone dimer from the endophytic fungus *Talaromyces* sp. associated with *Duguetia stelechantha* (Diels) R. E. Fries. J. Braz. Chem. Soc. doi: 10.5935/0103-5053.20130104

[ref37] Kuml D. Dethoup T. Buttachon S. Singburaudom N. Silva A. M. S. Kijjoa A. (2014). Spiculisporic acid E, a new spiculisporic acid derivative and ergosterol derivatives from the marine-sponge associated fungus *Talaromyces trachyspermus* (KUFA 0021). Nat. Prod. Commun. 9, 1147–1150. doi: 10.1177/1934578X1400900822, PMID: 25233594

[ref38] Küppers L. Ebrahim W. El-Neketi M. Özkaya F. Mándi A. Kurtán T.. (2017). Lactones from the sponge-derived fungus *Talaromyces rugulosus*. Mar. Drugs 15, 359. doi: 10.3390/md15110359, PMID: 29135916 PMC5706048

[ref39] Li H. Huang H. Shao C. Huang H. Jiang J. Zhu X.. (2011). Cytotoxic norsesquiterpene peroxides from the endophytic fungus *Talaromyces flavus* isolated from the mangrove plant *Sonneratia apetala*. J. Nat. Prod. 74, 1230–1235. doi: 10.1021/np200164k, PMID: 21545109

[ref40] Li H.-L. Li X.-M. Liu H. Meng L.-H. Wang B.-G. (2016). Two new diphenylketones and a new xanthone from *Talaromyces islandicus* EN-501, an endophytic fungus derived from the marine red alga *Laurencia okamurai*. Mar. Drugs 14. doi: 10.3390/md14120223, PMID: 27941601 PMC5192460

[ref41] Li X.-F. Yang Z.-D. Yang X. Yang L.-J. Yao X.-J. Shu Z.-M. (2021). Wortmannine H, a phenylpentenol isolated from an endophytic fungus, *Talaromyces wortmannii* LGT-4. Nat. Prod. Res. 35, 3204–3209. doi: 10.1080/14786419.2019.1690488, PMID: 31711315

[ref42] Liu F. Cai X.-L. Yang H. Xia X.-K. Guo Z.-Y. Yuan J.. (2010). The bioactive metabolites of the mangrove endophytic fungus *Talaromyces* sp. ZH-154 isolated from *Kandelia candel* (L.) Druce. Planta Med. 76, 185–189. doi: 10.1055/s-0029-1186047, PMID: 19670161

[ref43] Liu W.-C. Yang F. Zhang R. Shi X. Lu X.-H. Luan Y.-S.. (2016). Production of polyketides with anthelmintic activity by the fungus *Talaromyces wortmannii* using one strain-many compounds (OSMAC) method. Phytochem. Lett. 18, 157–161. doi: 10.1016/j.phytol.2016.10.006

[ref44] Luo Y. Lu X. Bi W. Liu F. Gao W. (2016). *Talaromyces rubrifaciens*, a new species discovered from heating, ventilation and air conditioning systems in China. Mycologia 108, 773–779. doi: 10.3852/15-233, PMID: 27055570

[ref45] Ma M. Yi W. Qin L. Lian X.-Y. Zhang Z. (2022). Talaromydien a and talaroisocoumarin A, new metabolites from the marine-sourced fungus *Talaromyces* sp. ZZ1616. Nat. Prod. Res. 36, 460–465. doi: 10.1080/14786419.2020.1779265, PMID: 34967248

[ref46] Matsuda Y. Abe I. (2020). Fungal meroterpenoids. Compr. Nat. Prod. III, 445–478. doi: 10.1016/B978-0-12-409547-2.14663-3 26497360

[ref47] Méndez-Líter J. A. Tundidor I. Nieto-Domínguez M. de Toro B. F. González Santana A. de Eugenio L. I.. (2019). Transglycosylation products generated by *Talaromyces amestolkiae* GH3 β-glucosidases: effect of hydroxytyrosol, vanillin and its glucosides on breast cancer cells. Microb. Cell Fact. 18, 97. doi: 10.1186/s12934-019-1147-4, PMID: 31151435 PMC6544938

[ref48] Nam I. H. Murugesan K. Ryu J. Kim J. H. (2019). Arsenic (As) removal using *Talaromyces* sp. KM-31 isolated from as-contaminated mine soil. Fortschr. Mineral. 9, 568. doi: 10.3390/min9100568

[ref49] Naraghi L. Heydari A. Rezaee S. Razavi M. (2012). Biocontrol agent *Talaromyces flavus* stimulates the growth of cotton and potato. J. Plant Growth Regul. 31, 471–477. doi: 10.1007/s00344-011-9256-2

[ref50] Ngokpol S. Suwakulsiri W. Sureram S. Lirdprapamongkol K. Aree T. Wiyakrutta S.. (2015). Drimane sesquiterpene-conjugated amino acids from a marine isolate of the fungus *Talaromyces minioluteus* (Penicillium Minioluteum). Mar. Drugs 13, 3567–3580. doi: 10.3390/md13063567, PMID: 26058010 PMC4483645

[ref51] Nicoletti R. Vinale F. (2018). Bioactive compounds from marine-derived *Aspergillus*, *Penicillium*, *Talaromyces* and *Trichoderma* species. Mar. Drugs 16, 408. doi: 10.3390/md16110408, PMID: 30373096 PMC6267100

[ref52] Nie Y. Liu Y. Yang W. Li Y. Xu M. Lei X.. (2019). Bioactive secondary metabolites from the fungus *Talaromyces* sp. isolated from coral *Porites pukoensis*. Mycosystema 38, 585–593. doi: 10.13346/j.mycosystema.180227

[ref53] Noinart J. Buttachon S. Dethoup T. Gales L. Pereira J. A. Urbatzka R.. (2017). A new ergosterol analog, a new bis-anthraquinone and anti-obesity activity of anthraquinones from the marine sponge-associated fungus *Talaromyces stipitatus* KUFA 0207. Mar. Drugs 15, E139. doi: 10.3390/md15050139, PMID: 28509846 PMC5450545

[ref54] Palem P. P. C. Kuriakose G. C. Jayabaskaran C. (2015). An *Endophytic fungus*, *Talaromyces radicus*, isolated from *Catharanthus roseus*, produces vincristine and vinblastine, which induce apoptotic cell death. PLoS One 10:e0144476. doi: 10.1371/journal.pone.0144476, PMID: 26697875 PMC4689362

[ref55] Pandit S. G. Puttananjaih M. H. Harohally N. V. Dhale M. A. (2018). Functional attributes of a new molecule-2-hydroxymethyl-benzoic acid 2′-hydroxy-tetradecyl ester isolated from *Talaromyces purpureogenus* CFRM02. Food Chem. 255, 89–96. doi: 10.1016/j.foodchem.2018.02.034, PMID: 29571503

[ref56] Ren J. Ding S.-S. Zhu A. Cao F. Zhu H.-J. (2017). Bioactive azaphilone derivatives from the fungus *Talaromyces aculeatus*. J. Nat. Prod. 80, 2199–2203. doi: 10.1021/acs.jnatprod.7b00032, PMID: 28749670

[ref57] Reyes B. A. S. Dufourt E. C. Ross J. Warner M. J. Tanquilut N. C. Leung A. B. (2018). Selected phyto and marine bioactive compounds: alternatives for the treatment of type 2 diabetes. Stud. Natl. Prod. Chem. 111–143. doi: 10.1016/B978-0-444-64068-0.00004-8

[ref58] Richardson M. Khosla C. (1999). “1.18- Structure, function, and engineering of bacterial aromatic Polyketide synthases,” in Comprehensive Natural Products Chemistry. eds. Barton S. D. Nakanishi K. Meth-Cohn O. (Pergamon: Oxford), 473–494.

[ref59] Sakai K. Asami Y. Chiba T. Suga T. Nonaka K. Iwatsuki M.. (2018). Oxoberkedienoic acid: a new octadienoic acid derivative isolated from *Talaromyces verruculosus* using a chemical screening system. J. Gen. Appl. Microbiol. 64, 136–138. doi: 10.2323/jgam.2017.09.001, PMID: 29553054

[ref60] Sparkman O. D. Penton Z. Kitson F. G. (2011). Gas Chromatography and Mass Spectrometry: A Practical Guide. 2nd Edn. Boston, MA: Elsevier.

[ref61] Uzma F. Mohan C. D. Hashem A. Konappa N. M. Rangappa S. Kamath P. V.. (2018). Endophytic fungi—alternative sources of cytotoxic compounds: a review. Front. Pharmacol. 9:309. doi: 10.3389/fphar.2018.00309, PMID: 29755344 PMC5932204

[ref62] Vasil I. K. Constabel F. Bogorad L. Schell J. S. (1984). Cell Culture and Somatic Cell Genetics of Plants. Orlando, FL: Academic Press.

[ref63] Venkatachalam M. Magalon H. Dufossé L. Fouillaud M. (2018). Production of pigments from the tropical marine-derived fungi *Talaromyces albobiverticillius*: new resources for natural red-colored metabolites. J. Food Compos. Anal. 70, 35–48. doi: 10.1016/j.jfca.2018.03.007

[ref64] Vinale F. Nicoletti R. Lacatena F. Marra R. Sacco A. Lombardi N.. (2017). Secondary metabolites from the endophytic fungus *Talaromyces pinophilus*. Nat. Prod. Res. 31, 1778–1785. doi: 10.1080/14786419.2017.1290624, PMID: 28278635

[ref65] Visagie C. M. Yilmaz N. Frisvad J. C. Houbraken J. Seifert K. A. Samson R. A.. (2015). Five new *Talaromyces* species with ampulliform-like phialides and globose rough walled conidia resembling *T. verruculosus*. Mycoscience 56, 486–502. doi: 10.1016/j.myc.2015.02.005

[ref66] Wang N. Qiu Y. Xiao T. Wang J. Chen Y. Xu X.. (2019). Comparative studies on Pb(II) biosorption with three spongy microbe-based biosorbents: high performance, selectivity and application. J. Hazard. Mater. 373, 39–49. doi: 10.1016/j.jhazmat.2019.03.056, PMID: 30901684

[ref67] Wang W. Wan X. Liu J. Wang J. Zhu H. Chen C.. (2018). Two new terpenoids from *Talaromyces purpurogenus*. Mar. Drugs 16, 150. doi: 10.3390/md16050150, PMID: 29724060 PMC5983281

[ref68] Waters D. M. Murray P. G. Ryan L. A. Arendt E. K. Tuohy M. G. (2010). *Talaromyces emersonii* thermostable enzyme systems and their applications in wheat baking systems. J. Agric. Food Chem. 58, 7415–7422. doi: 10.1021/jf100737v, PMID: 20496912

[ref69] West R. R. Ness J. V. Varming A. M. Rassing B. Biggs S. Gasper S.. (1996). ZG-1494α, a novel platelet-activating factor acetyltransferase inhibitor from *Penicillium rubrum*, isolation, structure elucidation and biological activity. J. Antibiot. 49, 967–973. doi: 10.7164/antibiotics.49.967, PMID: 8968388

[ref70] Xian H. Tang W. A. L. Li D. (2011). Cloning and bioinformatics analysis of chitinase gene from mycoparasitic *Talaromyces flavus*. J. Agric. Biotechnol. 9, 1089–1098. doi: 10.3969/j.issn.16747968.2011.06.016

[ref71] Xie X.-S. Fang X.-W. Huang R. Zhang S.-P. Wei H.-X. Wu S.-H. (2016). A new dimeric anthraquinone from endophytic *Talaromyces* sp. YE3016. Nat. Prod. Res. 30, 1706–1711. doi: 10.1080/14786419.2015.1136888, PMID: 26815015

[ref72] Yang H. Li F. Ji N. (2016). Alkaloids from an algicolous strain of *Talaromyces* sp. Chin. J. Ocean. Limnol. 34, 367–371. doi: 10.1007/s00343-015-4316-2

[ref73] Yang Z.-D. Zhang X.-D. Yang X. Yao X.-J. Shu Z.-M. (2021). A norbisabolane and an arabitol benzoate from *Talaromyces marneffei*, an endophytic fungus of *Epilobium angustifolium*. Fitoterapia 153:104948. doi: 10.1016/j.fitote.2021.104948, PMID: 34087409

[ref74] Yuan W.-H. Teng M.-T. Sun S.-S. Ma L. Yuan B. Ren Q.. (2018). Active metabolites from endolichenic fungus *Talaromyces* sp. Chem. Biodivers 15:e1800371. doi: 10.1002/cbdv.201800371, PMID: 30198640

[ref75] Zang Y. Genta-Jouve G. Escargueil A. E. Larsen A. K. Guedon L. Nay B.. (2016). Antimicrobial oligophenalenone dimers from the soil fungus *Talaromyces stipitatus*. J. Nat. Prod. 79, 2991–2996. doi: 10.1021/acs.jnatprod.6b00458, PMID: 27966935

[ref76] Zhai M.-M. Niu H.-T. Li J. Xiao H. Shi Y.-P. Di D.-L.. (2015). Talaromycolides A–C, novel phenyl-substituted phthalides isolated from the green Chinese onion-derived fungus *Talaromyces pinophilus* AF-02. J. Agric. Food Chem. 63, 9558–9564. doi: 10.1021/acs.jafc.5b04296, PMID: 26466717

[ref77] Zhang M. Deng Y. Liu F. Zheng M. Liang Y. Sun W.. (2021). Five undescribed steroids from *Talaromyces stipitatus* and their cytotoxic activities against hepatoma cell lines. Phytochemistry 189:112816. doi: 10.1016/j.phytochem.2021.112816, PMID: 34087503

[ref78] Zhang M. Li Q. Li S. Deng Y. Yu M. Liu J.. (2022a). An unprecedented ergostane with a 6/6/5 tricyclic 13(14 → 8)abeo-8,14-seco skeleton from *Talaromyces adpressus*. Bioorg. Chem. 127:105943. doi: 10.1016/j.bioorg.2022.105943, PMID: 35717801

[ref79] Zhang K. Zhang X. Lin R. Yang H. Song F. Xu X.. (2022b). New secondary metabolites from the marine-derived fungus *Talaromyces mangshanicus* BTBU20211089. Mar. Drugs 20, 79. doi: 10.3390/md20020079, PMID: 35200609 PMC8879399

[ref80] Zhang Y.-H. Zhao Y.-J. Qi L. Du H.-F. Cao F. Wang C.-Y. (2022c). Talasteroid, a new withanolide from the marine-derived fungus *Talaromyces stollii*. Nat. Prod. Res. 1–7. doi: 10.1080/14786419.2022.2070747, PMID: 35476591

[ref81] Zhao J. Liu Z. Sun S. Liu Y. (2020). Investigation on secondary metabolites of endophytic fungus *Talaromyces purpurogenus* hosted in Tylophora ovate. China J. Chin. Mater. Med. 6, 1368–1373. doi: 10.19540/j.cnki.cjcmm 32281350

[ref82] Zhao W.-T. Shi X. Xian P.-J. Feng Z. Yang J. Yang X.-L. (2021a). A new fusicoccane diterpene and a new polyene from the plant endophytic fungus *Talaromyces pinophilus* and their antimicrobial activities. Nat. Prod. Res. 35, 124–130. doi: 10.1080/14786419.2019.1616727, PMID: 31140306

[ref83] Zhao Y. Sun C. Huang L. Zhang X. Zhang G. Che Q.. (2021b). Talarodrides A-F, nonadrides from the antarctic sponge-derived fungus *Talaromyces* sp. HDN1820200. J. Nat. Prod. 84, 3011–3019. doi: 10.1021/acs.jnatprod.1c00203, PMID: 34842422

[ref84] Zhao J.-Y. Wang X.-J. Liu Z. Meng F.-X. Sun S.-F. Ye F.. (2019a). Nonadride and Spirocyclic anhydride derivatives from the plant endophytic fungus *Talaromyces purpurogenus*. J. Nat. Prod. 82, 2953–2962. doi: 10.1021/acs.jnatprod.9b00210, PMID: 31710490

[ref85] Zhao J.-W. Yang Z.-D. Zhou S.-Y. Yang L.-J. Sun J.-H. Yao X.-J.. (2019b). Wortmannine F and G, two new pyranones from *Talaromyces wortmannii* LGT-4, the endophytic fungus of *Tripterygium wilfordii*. Phytochem. Lett. 29, 115–118. doi: 10.1016/j.phytol.2018.11.023

[ref86] Zotchev S. B. (2013). “Alkaloids from marine bacteria,” in Advances in Botanical Research (Elsevier), 301–333. doi: 10.1016/B978-0-12-408061-4.00011-0

